# Proanthocyanidin Interferes with Intrinsic Antibiotic Resistance Mechanisms of Gram‐Negative Bacteria

**DOI:** 10.1002/advs.201802333

**Published:** 2019-05-28

**Authors:** Vimal B. Maisuria, Mira Okshevsky, Eric Déziel, Nathalie Tufenkji

**Affiliations:** ^1^ Department of Chemical Engineering McGill University 3610 University Street Montreal Quebec H3A 0C5 Canada; ^2^ INRS‐Institut Armand‐Frappier 531 boul. des Prairies Laval Québec H7V 1B7 Canada

**Keywords:** anti‐biofilm, antimicrobial, efflux pump, multidrug resistance, potentiation

## Abstract

Antibiotic resistance is spreading at an alarming rate among pathogenic bacteria in both medicine and agriculture. Interfering with the intrinsic resistance mechanisms displayed by pathogenic bacteria has the potential to make antibiotics more effective and decrease the spread of acquired antibiotic resistance. Here, it is demonstrated that cranberry proanthocyanidin (cPAC) prevents the evolution of resistance to tetracycline in *Escherichia coli* and *Pseudomonas aeruginosa*, rescues antibiotic efficacy against antibiotic‐exposed cells, and represses biofilm formation. It is shown that cPAC has a potentiating effect, both in vitro and in vivo, on a broad range of antibiotic classes against pathogenic *E. coli*, *Proteus mirabilis*, and *P. aeruginosa*. Evidence that cPAC acts by repressing two antibiotic resistance mechanisms, selective membrane permeability and multidrug efflux pumps, is presented. Failure of cPAC to potentiate antibiotics against efflux pump‐defective mutants demonstrates that efflux interference is essential for potentiation. The use of cPAC to potentiate antibiotics and mitigate the development of resistance could improve treatment outcomes and help combat the growing threat of antibiotic resistance.

## 1 Introduction

The global spread of antibiotic resistance is undermining decades of progress in fighting bacterial infections. Due to the overuse of antibiotics in medicine and agriculture, we are on the cusp of returning to a pre‐antibiotic era in which minor infections can once again become deadly. Countering the fall in antibiotic efficacy by improving the effectiveness of currently available antibiotics is therefore an important goal. Antibiotic efficacy is limited by the expression of intrinsic tolerance mechanisms such as production of antibiotic‐tolerant and/or persister cells, or formation of biofilm, and emergence of antibiotic‐resistant pathogens recalcitrant to treatment due to acquired resistance. Here, we report that a purified cranberry proanthocyanidin (cPAC) fraction potentiates the activity of a broad range of antibiotic classes against the opportunistic pathogens *Escherichia coli*, *Proteus mirabilis*, and *Pseudomonas aeruginosa*. cPAC was an effective potentiator against diverse bacterial lifestyles normally tolerant to antibiotics, such as biofilm bacteria, dormant cells, and in experimental models of chronic infections. Remarkably, when combined with tetracycline, cPAC was able to completely prevent the evolution of resistance in *E. coli* and *P. aeruginosa*. These results suggest that in combination with antibiotic therapy, cPAC has the potential to decrease the spread of antibiotic resistance and prolong the effectiveness of currently available drugs.

Opportunistic pathogens colonize surfaces in healthcare settings, on indwelling medical devices, and on living tissues, leading to infections that must be treated with antibiotics. Two major factors complicate the effectiveness of antibiotic treatments: i) antibiotic‐resistant bacteria and ii) the formation of antibiotic‐tolerant biofilms.^1^ The latter sessile bacteria can withstand antibiotic doses up to thousands of times higher than their planktonic counterparts,^2^, ^3^, ^4^ due largely to the presence of tolerant and persister cells that remain dormant within the biofilm, ready to reinitiate growth when antibiotic concentrations decrease. Prolonged antibiotic treatment and high antibiotic doses necessitated by the recalcitrant nature of such infections put patients' health at risk and create a strong selective pressure for the evolution of antibiotic resistance.^2^, ^3^, ^5^ In order to decrease the occurrence of antibiotic resistance and mitigate negative side effects brought on by high antibiotic doses, novel approaches to enhance the effectiveness of currently available antibiotics represent an attractive alternative to the search for new antibiotics. Exploiting natural molecules with antibiotic‐potentiating activities provides one such opportunity.

The American cranberry (*Vaccinium macrocarpon* L.) fruit and its derivatives have long been anecdotally reported as a natural remedy for urinary tract infections.^6^, ^7^ cPAC are condensed tannins that can hinder bacterial attachment to cellular or biomaterial surfaces,^8^, ^9^, ^10^, ^11^ impair bacterial motility,^12^, ^13^, ^14^, ^15^, ^16^, ^17^ induce a state of iron limitation,^18^ and interfere with quorum sensing.^19^ Studies suggest that consumption of cranberry can prevent bacterial infections,^20^, ^21^, ^22^, ^23^, ^24^, ^25^ and one reports the combined effect of bulk cranberry derivatives (devoid of the proanthocyanidin fraction) and β‐lactam antibiotics against a Gram‐positive bacterium.^26^ It has also been suggested that cPAC can inhibit biofilm formation and potentiate gentamicin against *P. aeruginosa*;^17^ however, the potential for cPAC to interfere with the evolution of resistance to antibiotics or rescue the effectiveness of antibiotics has never been investigated. Furthermore, no studies have explored the activity spectrum or the molecular mode of action of specific cranberry‐derived fractions such as proanthocyanidins for the treatment of bacterial infections. Here, we report the ability of cPAC to thwart the evolution of resistance to tetracycline in *E. coli* and *P. aeruginosa*, and demonstrate the broad spectrum, antibiotic‐potentiating activity of cPAC against various pathogenic Gram‐negative bacteria, both in vitro and in vivo. We also show that cPAC is able to repress two important intrinsic antibiotic resistance mechanisms: selective membrane permeability and multidrug efflux pump activity.

## 2 Results

### 2.1 cPAC Potentiates a Broad Range of Antibiotics In Vitro

**Figure 1:**
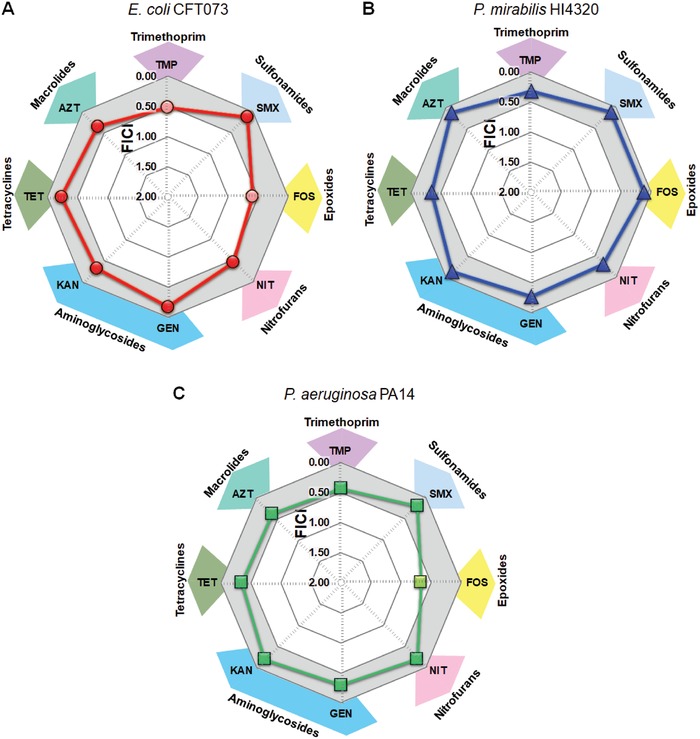
Potentiating interaction of cPAC with antibiotic results in growth inhibition. MICs were determined for the combination of cPAC with each antibiotic in vitro. Fractional inhibitory concentration index (FICI) for each combination are shown for A) *E. coli* CFT073, B) *P. mirabilis* HI4320, and C) *P. aeruginosa* PA14. A FICI of ≤0.5 is indicated by the gray shaded area. TMP: trimethoprim; SMX: sulfamethoxazole; FOS: fosfomycin; NIT: nitrofurantoin; GEN: gentamicin; KAN: kanamycin; TET: tetracycline; AZT: azithromycin.

We conducted screening assays with cPAC in combination with several classes of clinically approved antibiotics belonging to the World Health Organization's list of essential medications.^27^ Checkerboard microdilution analysis was performed using pathogenic strains of *E. coli*, *P. mirabilis*, and *P. aeruginosa*. We determined whether cPAC was able to potentiate the effectiveness of antibiotics by calculating the fractional inhibitory concentration index (FICI). **Figure** **1**A–C shows positive and/or negative interactions between cPAC and antibiotics against bacterial pathogens. FICI values of ≤0.5 (indicated by the gray zone) demonstrate that cPAC potentiated the effectiveness of sulfamethoxazole (SMX), nitrofurantoin (NIT), gentamicin (GEN), kanamycin (KAN), tetracycline (TET), and azithromycin (AZT) to inhibit the growth of *E. coli* CFT073, *P. mirabilis* HI4320, and *P. aeruginosa* PA14 using up to 98% less antibiotic than that required in the absence of cPAC. cPAC also potentiated trimethoprim (TMP) and fosfomycin (FOS) activities to inhibit the growth of *P. mirabilis* HI4320; 81% and 98% less antibiotic were needed, respectively, than in the absence of cPAC. In the case of *P. aeruginosa* strain PAO1, cPAC enhanced the efficiency of the antibiotics SMX, FOS, NIT, GEN, KAN, and AZT (Figure S1A, Supporting Information). The fact that cPAC potentiates a given antibiotic against one strain but not another (e.g., cPAC potentiates FOS against *P. mirabilis* HI4320 but not against *P. aeruginosa* PA14 or *E. coli* CFT073) provides evidence that the effect is specific and that cPAC is not simply inactivating the antibiotic. It is not surprising that the FICI values for the two *P. aeruginosa* strains were different for some antibiotics as they had different MIC_antibiotic_ values.^28^

At concentrations required to potentiate antibiotic efficacy, cPAC alone had no detectable growth inhibition activity against all four pathogenic strains (Figures S1B and S2A–C, Supporting Information). Given the ability of cPAC to potentiate TMP or SMX alone, we investigated the interaction of cPAC with co‐trimoxazole (the combination of SMX and TMP, commonly used to treat urinary tract infections and bacterial dysentery^27^). cPAC enhanced the synergistic efficacy of co‐trimoxazole, reducing the MIC up to 64‐fold against *P. mirabilis* HI4320. In the case of *P. aeruginosa* PA14, combination of cPAC with co‐trimoxazole decreased the MIC by 32‐fold, which is significantly more effective than the potentiating combinations of cPAC with TMP or SMX alone (Figure S3, Supporting Information). The fact that cPAC potentiates antibiotics, but does not act as an antibiotic on its own, suggests that treatment with cPAC is unlikely to create selective pressure for the evolution of resistance.

### 2.2 cPAC Prevents Re‐Activation of Antibiotic‐Exposed Cells

**Figure 2:**
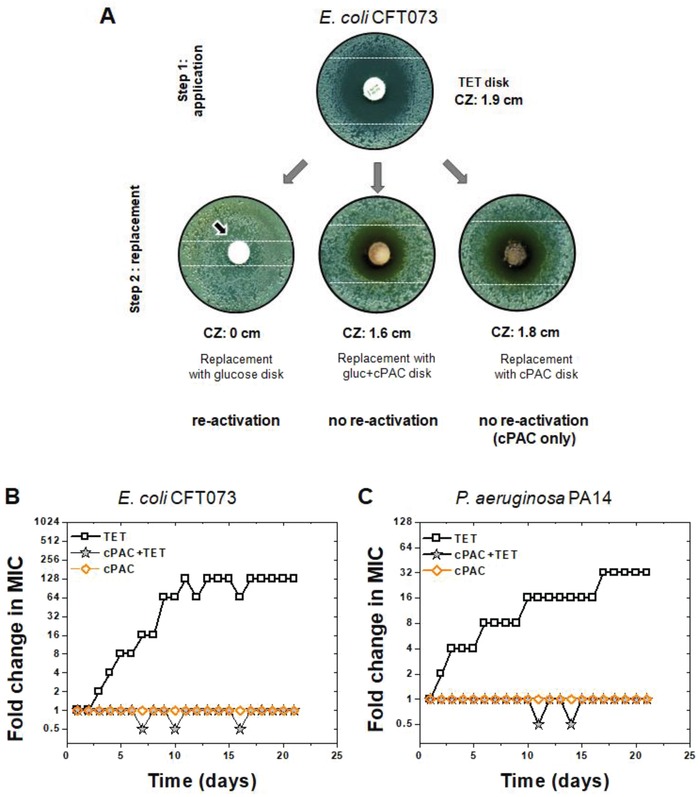
Synergistic effect of cPAC with TET for the inhibition of growth re‐activation of antibiotic‐exposed cells and prevention of the evolution of resistance. A) Detection of growth re‐activation of antibiotic‐exposed *E. coli* CFT073 cells using a modified disk‐diffusion assay. Step 1: a TET antibiotic disk was placed on top of MHB‐II agar. The dashed lines mark the diameter of the clear zone surrounding the TET disk. Step 2: the TET disks are replaced with a glucose alone, cPAC+glucose, or cPAC alone disk on the MHB‐II agar plate. The diameter of the clear zone (CZ) and no colony formation inside the clear zone surrounding the cPAC+glucose disk indicate no re‐activation of antibiotic‐exposed cells after disk replacement at Step 2 and colonies inside the inhibition zone (indicated by black arrow) surrounding the glucose disk indicate re‐activation of antibiotic‐exposed cells. The cPAC‐only disk prevented re‐activation of growth, most likely because of synergy with TET remnants. The images shown are only representative images of three independent experiments. Disk diameter: 6 mm. Emergence of antibiotic resistance in B) *E. coli* CFT073 and C) *P. aeruginosa* PA14 during 21 serial passages in the presence of sub‐MIC levels of TET compared to 400 µg mL^−1^ cPAC alone or its combination with 100 µg mL^−1^ cPAC.

To investigate the inhibitory activity of cPAC against antibiotic‐exposed bacteria, a modified disk‐diffusion test was performed. **Figure** **2**A shows that following treatment with the bacteriostatic antibiotic tetracycline (Step 1: application of TET antibiotic disk), bacteria in the growth‐inhibition zone were able to recover when a new disk impregnated with glucose replaced the TET disk (at Step 2). An analysis of bacterial re‐activation based on the occurrence of colonies inside a typical inhibition/clear zone shows that the degree to which cells “re‐activate” to form colonies differs depending on the presence or absence of cPAC. Replacement with a glucose‐only disk (at Step 2) enhanced the re‐activation of cells (i.e., bacterial lawn in previous inhibition zone), while a disk with a combination of cPAC and glucose showed no re‐activation of antibiotic‐exposed cells. There were no colonies observed close to the cPAC‐only disk, and the size of the clear zone with the cPAC‐only disk (at Step 2) was similar to the TET disk (Step 1) and the glucose+cPAC disk (Step 2). Since cPAC alone did not inhibit bacterial growth of this, or any other tested strains (Figures S1B,C and S2A–F, Supporting Information), it is probable that the clear zones around the cPAC‐only and the glucose+cPAC disks result from synergy of cPAC with TET remnants, which is in agreement with a slightly smaller diameter, since lower concentrations of cPAC and TET should be present at the distal edge of the zone. Similar effects were observed with minocycline (MIN; Figure S4, Supporting Information) against *E. coli* CFT073. These results suggest that cPAC can prolong the efficacy of remnant antibiotic against antibiotic‐exposed cells even after treatment has ceased.

### 2.3 cPAC Thwarts Evolution of Antibiotic Resistance

To understand the role of cPAC in effectively inhibiting the re‐activation of antibiotic‐exposed cells, we analyzed the ability of cPAC to suppress evolution of resistance in *E. coli* CFT073 and *P. aeruginosa* PA14. As shown in Figure 2B,C, sequential passaging on TET alone for 21 days resulted in 128‐fold and 32‐fold increases in MIC for *E. coli* CFT073 and *P. aeruginosa* PA14, respectively, while cPAC prevented the evolution of resistance in both strains when co‐administered with TET. cPAC alone did not promote resistance in either strain. This result shows that cPAC can suppress the emergence of antibiotic resistance in bacteria.

### 2.4 cPAC Potentiates In Vivo Activity of SMX

**Figure 3:**
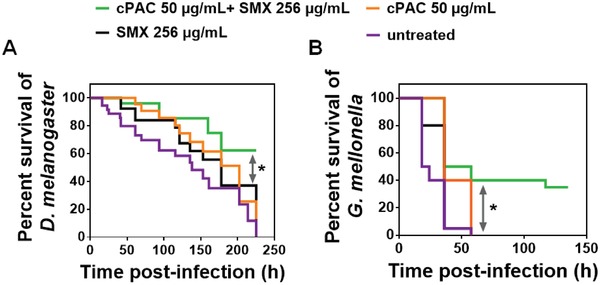
In vivo synergistic effect of cPAC with antibiotic for the protection of insect models. A) In vivo synergy between sulfamethoxazole (SMX) and cPAC was tested in a *D. melanogaster* fly feeding model. Flies (*N* = 30 per experimental group) were infected orally with *P. aeruginosa* PA14 cells and maintained on agar containing SMX in combination with cPAC. Results represent measurements from experiments performed in triplicate (*, *P* < 0.05, log‐rank (Mantel–Cox) test). B) In vivo synergy between SMX and cPAC was tested in a *G. mellonella* larvae infection model. *G. mellonella* larvae were infected with a lethal dose of *P. aeruginosa* PA14 cells. These infected *G. mellonella* larvae (*N* = 20 per experimental group) were injected a second time at the same infection site with cPAC or SMX, alone or in combination at 3 h post. Results represent measurements from two independent experiments performed in duplicate (*, *P* < 0.005, log‐rank (Mantel–Cox) test).

Our next goal was to investigate the potential of cPAC to enhance the efficacy of antibiotics against bacterial infections in vivo. To this end, we used the model host *Drosophila melanogaster* infected with *P. aeruginosa* PA14, in which cPAC or SMX was administered alone or in combination. The median survival of the insects following infection was 138 h in the absence of treatment, but more than 225 h when cPAC and SMX were combined (**Figure** **3**A). Survival of flies following combination therapy was significantly (χ^2^ = 3.88, d*f* = 1, *P* < 0.05) higher than survival with SMX treatment alone. The median survival time of infected *D. melanogaster* treated with cPAC alone or SMX alone was 202 and 178 h, respectively (Figure 3A), which is not significantly (*P* > 0.05) different from the survival of untreated flies infected with *P. aeruginosa* PA14. The survival of uninfected *D. melanogaster* was similar to treatment with cPAC or SMX alone (Figure S5A, Supporting Information), which indicates that cPAC (at 50 µg mL^−1^) or SMX (at 256 µg mL^−1^) alone is safe for this animal.

To confirm that cPAC is able to potentiate antibiotics in more than one host, we also used the greater wax moth (*Galleria mellonella*) larvae killing model, in which cPAC or SMX was administered alone, or in combination, to larvae infected with a lethal dose of *P. aeruginosa* PA14. The median survival of infected and untreated larvae was 21 h, but increased to 47 h with the cPAC and SMX combination treatment. This is a significantly (χ^2^ = 14.3, d*f* = 1, *P* < 0.005) longer survival time than the 36 h median survival time with SMX treatment alone (Figure 3B). The median survival of infected larvae with cPAC treatment alone was similar to the treatment of SMX alone (Figure 3B), which is in contrast to cPAC's inability to act as an antibiotic at this concentration in vitro, or in the fly feeding model. As with the *D. melanogaster* feeding assay, there was no difference in survival curves of uninfected *G. mellonella* larvae with or without treatment of cPAC or SMX alone (Figure S5B, Supporting Information). These results confirm that cPAC potentiates the activity of SMX in vivo, at the tested concentration of 50 µg mL^−1^ cPAC.

### 2.5 cPAC in Combination with an Antibiotic Impairs Biofilm Formation

**Figure 4:**
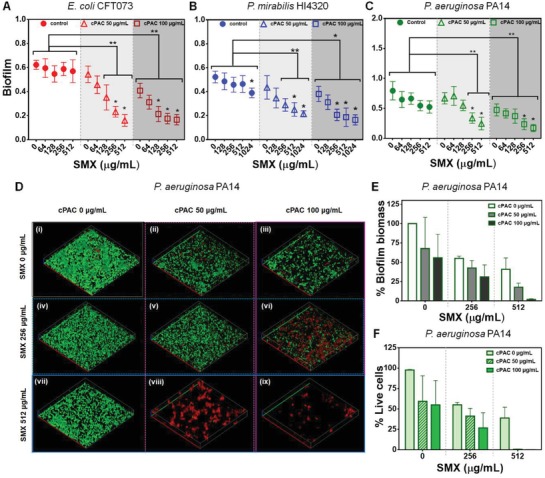
Effect of cPAC alone and in combination with SMX on biofilm formation of A) *E. coli* CFT073, B) *P. mirabilis* HI4320, and C) *P. aeruginosa* PA14. The graphs present normalized biofilm levels (OD_570_/cell OD_600_) at subinhibitory concentrations of SMX. Statistically significant differences are indicated for each sample treated with cPAC and SMX compared to the control (sample treated with the corresponding concentration of SMX alone) (**, *P* < 0.01; *, *P* < 0.05; two‐way ANOVA) and for samples treated with cPAC plus SMX compared to the sample treated with the same concentration of cPAC without SMX (*, *P* < 0.05; two‐way ANOVA). D) Confocal microscopy images of *P. aeruginosa* PA14 biofilms grown with or without cPAC and/or SMX at sub‐MICs. Green color cells represent viable cells with intact membranes and red color cells are dead cells. Each representative image was selected from experiments performed in triplicates. E) Biofilm biomass as a percentage of untreated biomass, and F) percentages of live cells present in each treatment were quantified from confocal microscopy images. In all confocal microscopy images, red and green axis lengths are 100 µm, and the blue *z*‐axis length is 10 µm.

To explore whether cPAC can impair the formation of biofilms during antibiotic treatment, monoculture bacterial biofilms were grown in microtiter plates in the presence of different concentrations of cPAC and SMX. As shown in **Figure** **4**A–C, cPAC decreased biofilm formation of *E. coli* CFT073, *P. mirabilis* HI4320, and *P. aeruginosa* PA14. cPAC alone at 100 µg mL^−1^ showed significant (*P* < 0.05) inhibition of monoculture biofilms, which is consistent with other studies showing that cPAC or cranberry extracts can decrease bacterial adhesion.^8^, ^9^, ^10^, ^11^ Importantly, cPAC in combination with SMX had a significant (*P* < 0.01) inhibitory effect on the development of biofilms in a dose‐dependent manner (Figure 4A–C; Figure S1D, Supporting Information). Untreated control biofilms were composed of viable cells (Figure 4D, in green) attached to the surface and forming dense microcolonies (Figure 4Di). SMX alone at tested concentrations had minimal effect on biofilms. However, treatment with cPAC and SMX in combination resulted in a decrease in the total density of attached biomass and viability when compared to untreated biofilms (Figure 4E,F). Interestingly, the majority of the bacterial biomass was dead when treated with 512 µg mL^−1^ SMX in combination with cPAC (Figure 4Dviii,ix). If we presume that the few cells left alive in the presence of 512 µg mL^−1^ SMX and 50 µg mL^−1^ cPAC are dormant, capable of reactivating when the antibiotic threat has passed and forming a new biofilm, then increasing the concentration of cPAC to 100 µg mL^−1^ was sufficient to eradicate these surviving cells. Because a combination of cPAC and SMX is more effective against biofilms than either compound is alone, these observations demonstrate that cPAC acts synergistically with SMX to reduce the ability of bacteria to form biofilms, and supports the hypothesis that cPAC contributes to the impairment of dormant antibiotic‐exposed cells.

### 2.6 Mechanisms by Which cPAC Potentiates Antibiotic Activity

**Figure 5:**
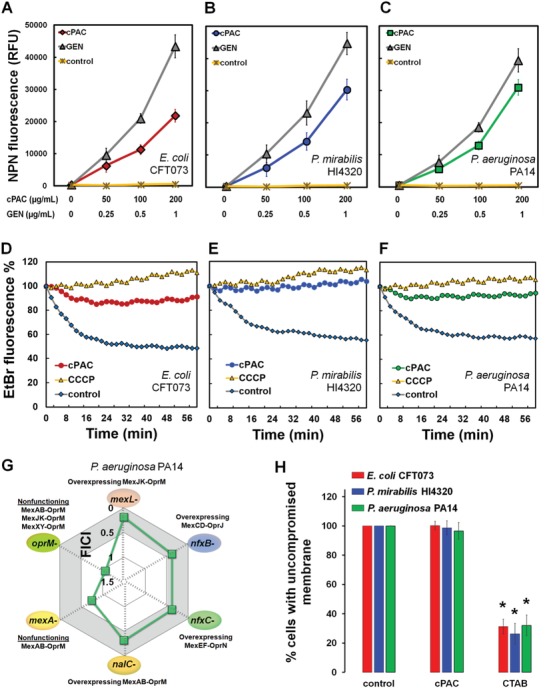
Mechanisms of antibiotic potentiation by cPAC. cPAC‐mediated NPN uptake in A) *E. coli* CFT073, B) *P. mirabilis* HI4320, and C) *P. aeruginosa* PA14. Bacterial cells were pretreated with cPAC or gentamicin (Gen) for NPN uptake. Control represents NPN fluorescence in the presence of only cPAC without bacteria. Inhibition of multidrug efflux pump by cPAC or the known efflux pump inhibitor CCCP in D) *E. coli* CFT073, E) *P. mirabilis* HI4320, and F) *P. aeruginosa* PA14. Control represents untreated bacteria. G) MICs were determined for the combination of cPAC with TET in vitro. FICI values for cPAC+TET combination are shown for efflux pump mutants of *P. aeruginosa* PA14: *mexL^−^* (overexpressing MexJK‐OprM), *nfxB^−^* (overexpressing MexCD‐OprJ), *nfxC^−^* (overexpressing MexEF‐OprN), *nalC^−^* (overexpressing MexAB‐OprM), *mexA^−^* (nonfunctioning MexAB‐OprM), and *oprM^−^* (nonfunctioning MexAB‐OprM, MexJK‐OprM, and MexXY‐OprM). A FICI index of ≤0.5 is indicated by the gray shaded area. H) Effect of cPAC on cell membrane integrity. Cells of each strain were pretreated separately with cPAC or the cell membrane disrupting agent CTAB. The ratio of green to red fluorescence was normalized to that of the untreated control and expressed as a percentage of the control. All assays were repeated independently three times (* *P* < 0.05; Student's *t‐*test).

To identify the mechanism(s) of action by which cPAC potentiates antibiotic activity, we quantified the changes in bacterial cell outer membrane permeability using 1‐*N*‐phenylnapthylamine (NPN) as an indicator, which revealed that cPAC increases outer membrane permeability of bacterial cells (**Figure** **5**A–C). Investigations into efflux pump activity using ethidium bromide (EtBr) as a fluorescent indicator substrate showed that, in contrast to the decay in fluorescence observed in untreated cells (Figure 5D–F), cells treated with cPAC, or the efflux pump inhibitor carbonyl cyanide *m*‐chlorophenylhydrazone (CCCP), remained fluorescent over time due to a failure to pump out EtBr (Figure 5D–F). These observations demonstrate that cPAC is able to inhibit multidrug resistance efflux pumps. To further investigate the interaction between cPAC and multidrug resistance efflux pumps, we employed a systematic checkerboard MIC analysis of *P. aeruginosa* PA14 efflux pump mutants. The overexpression of efflux pump systems causes increased resistance to antibiotics compared to wild‐type isolates while disruption of efflux pump protein components is associated with increased intracellular antibiotic accumulation and antibiotic susceptibility.^29^ We show here that the ability of cPAC to potentiate TET activity correlates with efflux pump activity. The mutant strains with nonfunctioning efflux pumps were more susceptible to TET compared to the wild‐type strain, such that addition of cPAC provided no further benefit for the potency of TET. Therefore, potentiation between cPAC and TET was not observed in efflux pump‐nonfunctioning mutants (Figure 5G). This lack of TET potentiation correlates well with TET potentiation by cPAC in the case of wild type *P. aeruginosa* PA14, which has functioning efflux pumps (Figure 1C). Interestingly, cPAC caused no significant damage to bacterial cell membranes (Figure 5H), compared to significant (*P* < 0.05) membrane disruption by cetyl trimethylammonium bromide (CTAB), a cell membrane–disrupting agent, suggesting that outer membrane permeabilization and target specific efflux pump inhibition are achieved by cPAC without altering cell membrane integrity.

### 2.7 cPAC Interacts with Efflux Pump Components In Silico

**Figure 6:**
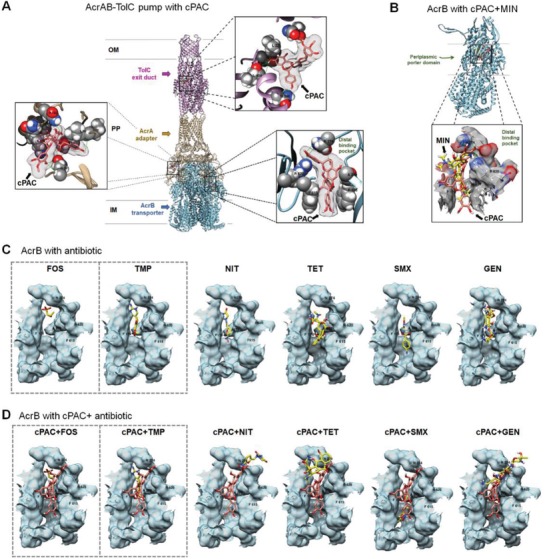
Molecular docking analysis of cPAC in the AcrAB–TolC efflux pump. A) Complete side view with ribbon representation of docked complexes of efflux pump proteins with A‐type cPAC molecule (a dimeric form of epicatechin), visualized along the Gram‐negative cell membrane plane (OM, outer membrane; PP, periplasmic space; and IM, inner membrane). The inset views show the electron density map (2*F*_0_–*F*_c_) of cPAC in binding sites of multidrug efflux pump exit duct, adapter, and transporter proteins. The amino acid residues around each binding site are depicted and all possible hydrogen bonds are shown using green lines. The ribbon representation of tripartite efflux pump components are color‐coded: TolC exit duct (pink), AcrA adapter (gold) and AcrB transporter (blue). B) The AcrB monomer with inset views shows docking of cPAC molecule (salmon) to the distal binding pocket in the co‐crystal structure of AcrB–MIN complex (yellow color carbons represent MIN molecule as found in the highest resolution crystal structure, PDB 4U8Y). C) Molecular docking analysis of different efflux pump substrates (yellow) binding at the distal binding pocket of AcrB transporter. D) The inset views show the optimum binding position of cPAC (salmon) and substrate (yellow) at the distal binding pocket of AcrB. The docking models of nonpotentiated antibiotics are highlighed with a dashed box.

To understand how cPAC is able to inhibit the activity of efflux pumps, we performed in silico docking analyses using the efflux pump protein complexes AcrAB–TolC of *E. coli*, and MexAB–OprM of *P. aeruginosa*, with the A‐type dimeric cPAC molecule as the test ligand. Ligand‐binding domains of AcrAB–TolC and MexAB–OprM efflux pump components exhibit sufficient space to accommodate the cPAC molecule with an average volume of 497.1 Å^3^ (Table S1, Supporting Information). We tested structural in silico interactions of cPAC with the exit duct, adapter, and transporter components of the efflux pumps (**Figure** **6**A; Figure S6A–G, Supporting Information). Molecular docking of cPAC with the exit duct complex predicts that cPAC favorably binds at the equatorial domain of the TolC exit duct (Figure 6A) and coiled‐coil domain of the OprM exit duct (Figure S6A–C, Supporting Information). The molecular docking analyses also predict that cPAC can form hydrogen bonds with amino acid residues (N274, A279, and R620) present in the distal substrate‐binding pocket of AcrB that are critical for antibiotic/substrate binding, transportation to the exit duct, and proper functioning of the entire efflux pump assembly (Figure 6A). cPAC showed the highest affinity for AcrB and MexB efflux pump components, with −9.9 and −9.6 kcal mol^−1^ binding energies, respectively (Table S1, Supporting Information).

A diverse range of antibiotics are pumped out by AcrAB–TolC. However, only the co‐crystal structures of MIN and doxorubicin binding to the active site of AcrB are reported.^30^ To compare the binding of cPAC to that of a well‐characterized efflux pump substrate, we docked cPAC to the distal binding pocket of the AcrB periplasmic porter domain co‐crystallized with MIN, and found that cPAC and MIN bound to the same furrow of the distal substrate binding pocket (Figure 6B). We separately examined the predicted docking of the AcrB substrates FOS, TMP, NIT, TET, SMX, and GEN in the absence and presence of cPAC (Figure 6C,D). All tested substrates bound to the AcrB–cPAC complex, specifically, in the same furrow of the distal binding pocket in which MIN binds (Figure 6C). Interestingly, the binding position of FOS and TMP was unaffected by the presence of cPAC (Figure 6D), which is in agreement with the inability of cPAC to potentiate FOS or TMP against *E. coli* in vitro (Figure 1A). In contrast, we observed different binding conformations of the potentiated antibiotics NIT, TET, SMX, and GEN in the presence of cPAC compared to that in the absence of cPAC (Figure 6C,D). This suggests that binding of cPAC in the distal binding pocket leads to efflux inhibition by interfering with the preferred binding position of the potentiated antibiotics. Finally, we confirmed that the binding conformation of cPAC in the distal binding pocket of AcrB is similar to that of known efflux pump inhibitors (Figure S7A–F, Supporting Information), which supports the hypothesis that cPAC acts as a potent efflux pump inhibitor.

## 3 Discussion

Tetracycline resistance is a well‐documented phenomenon caused by efflux pump activity and the evolution of acquired antibiotic resistance.^31^ We discovered that in the presence of cPAC, the acquisition of resistance in *E. coli* and *P. aeruginosa* following TET treatment is completely abrogated. cPAC's ability to interfere with intrinsic resistance mechanisms is therefore able to suppress the typically inevitable, long‐term evolution of acquired antibiotic resistance.

We have shown that cPAC potentiates the in vitro activity of a range of antibiotic classes against the opportunistic human pathogens *E. coli*, *P. mirabilis*, and *P. aeruginosa*. This potentiation also occurs in vivo, at least with the antibiotic SMX. cPAC is an especially promising antibiotic‐potentiating agent because it does not exhibit antimicrobial activity of its own. Agents that do not negatively affect the viability of bacterial pathogens are less likely to promote resistance than conventional antibiotics^32^, ^33^, ^34^ and are, therefore, especially well suited for combination treatment with antibiotics. Accordingly, we have not observed any evolution of resistance to cPAC.

Biofilms can lead to chronic bacterial infections and are commonly associated with antibiotic treatment failure.^35^, ^36^ The presence of persister and antibiotic‐tolerant cells is closely linked to biofilm formation,^5^ and also plays an important role in the recalcitrance of chronic infections. The antiquorum sensing activity of a cranberry proanthocyanidin fraction alone^19^ and in combination with ciprofloxacin^37^ has been reported. As quorum sensing is required for normal biofilm formation, this agrees with our observations that cPAC in combination with antibiotic substantially represses biofilm formation. Furthermore, we propose that it is the ability of cPAC to target not only actively metabolizing cells, but also dormant antibiotic‐exposed cells that enable cPAC to potentiate antibiotic efficacy against biofilms.

The intrinsic antibiotic resistance mechanisms of bacterial cells are naturally occurring phenomena found in bacterial species that complicate antibiotic treatments. For instance, *P. mirabilis* has intrinsic resistance to NIT and TET, and *P. aeruginosa* has intrinsic resistance to multiple antibiotic classes including aminoglycosides, β‐lactams, quinolones, and polymyxins.^38^, ^39^ The intrinsic antibiotic resistance mechanisms include selective outer membrane permeability, poor antibiotic transport, and active multidrug efflux.^40^, ^41^ Among resistance–nodulation–division family efflux pumps, AcrAB–TolC^42^, ^43^ and MexAB–OprM^44^ are well known for their importance in bacterial survival and intrinsic antibiotic resistance. Our in silico analysis predicts that the A‐type dimeric cPAC molecule (which is the most common terminating unit of the cPAC fraction^45^) can occupy the ligand‐binding pocket of the AcrB transporter in *E. coli* and MexB transporter of *P. aeruginosa*.^43^, ^44^, ^46^ Molecular docking calculations indicate that of all the antibiotics tested against *E. coli*, the two nonpotentiated antibiotics adopt the same position in the distal binding pocket in the presence and absence of cPAC. In contrast, the potentiated antibiotics are predicted to take a different binding position in the presence of cPAC, which would hinder their efflux from the cell. Exposure to cPAC enhanced membrane permeabilization and decreased efflux pump activity in all tested wild‐type bacteria in vitro. The failure of cPAC to potentiate antibiotic activity in efflux pump‐defective mutants supports a model where the specific effect cPAC has on efflux is essential for the observed antibiotic potentiating activity. Inactivation of efflux pump activity has previously been shown to have a negative impact on biofilm formation^47^ and pathogenicity^48^ in Gram‐negative bacteria. This is consistent with our observations that in the presence of cPAC, biofilm formation is decreased. Overall, this study provides a proof of concept and a starting point for investigating the molecular mechanism of the reported efflux pump inhibition in bacteria by cPAC.

Insect animal models have a relatively evolved antimicrobial defense system and are thus often used to generate information relevant to the mammalian infection process.^49^, ^50^ In both infection models used in this study, cPAC at a dose of 50 µg mL^−1^ was sufficient to potentiate the activity of the tested antibiotic and significantly increase survival rates of the animals during infection with *P. aeruginosa*. These in vivo studies provide a promising outlook for the potential future development of cPAC as an antibiotic‐potentiating agent in higher organisms; however, a few studies report on the safety of cPAC to human cells^51^ or its bioavailability and the rate of clearance in animals.^52^ Thus, further work is needed to verify the efficacy and safety of the combination treatment in an in vivo mammalian (e.g., mouse) model. Encouragingly, our data show that cPAC is able to enhance the efficacy of a broad spectrum of antibiotics. The ability to potentiate the action of antibiotics in a patient could improve treatment outcomes and hinder the emergence of antibiotic‐resistant infections.

## 4 Experimental Section

*Bacterial Strains, Growth Conditions, and Cranberry Proanthocyanidin*: Opportunistic bacterial pathogens were used in this study: *E. coli* strain CFT073 (ATCC 700928), *P. mirabilis* HI4320,^53^ *P. aeruginosa* PAO1 (ATCC 15692), and *P. aeruginosa* PA14 (UCBPP‐PA14).^54^ Mutant strains of *P. aeruginosa* PA14 were used in this study: *mexL^−^* (overexpressing MexJK‐OprM),^55^ *nfxB^−^* (overexpressing MexCD‐OprJ),^55^ *nfxC^−^* (overexpressing MexEF‐OprN),^56^ *nalC^−^* (overexpressing MexAB‐OprM),^55^ *mexA^−^* (nonfunctioning MexAB‐OprM),^55^ and *oprM^−^* (nonfunctioning MexAB‐OprM, MexXY‐OprM, and MexJK‐OprM).^55^ Pure stock cultures were maintained at −80 °C in 30% (v/v) frozen glycerol solution. Starter cultures were prepared by streaking frozen cultures onto lysogeny broth agar (LB broth contained 10 g L^−1^ tryptone, 5 g L^−1^ yeast extract, and 5 g L^−1^ NaCl, supplemented with 1.5% (w/v) agar (Fisher Scientific, ON, Canada)). After overnight incubation at 37 °C, a single colony was inoculated into 10 mL of Mueller–Hinton broth adjusted with Ca^2+^ and Mg^2+^ (MHB‐II; Oxoid, Fisher Scientific Canada) and the culture was incubated at 37 °C on an orbital shaker at 200 rpm for a time length specific to each experiment. LB broth was used for bacterial culture in all experiments unless otherwise specified. The purified cranberry‐derived proanthocyanidin (cPAC, 93% proanthocyanidins, 7% anthocyanins, and flavonoids monomers) was obtained from Ocean Spray Cranberries Inc. The supplier prepared the sample according to well‐established methods^11^, ^57^ by enriching from cranberry fruit juice extract. A dry powder of cPAC was solubilized in deionized water and sterilized by filtration (0.22 µm polyvinylidene fluoride (PVDF) membrane filter).

*Determination of MICs*: MICs were determined by preparing twofold serial dilutions of cPAC, and antibiotics in MHB‐II broth. A range of concentrations of the antibiotics (0.0003–1024 µg mL^−1^) was chosen due to their known potency against all four bacterial strains. Bacterial growth was assessed by i) monitoring the cell growth (observed as a pellet and turbidity) in the wells^58^ ii) monitoring the optical density of the cell suspension in each well at 600 nm (OD_600_), and iii) using the resazurin microtiter plate assay.^59^ Additional details can be found in the Supporting Information.

*In Vivo Infection Assay Using D. Melanogaster Flies*: Fruit flies (*D. melanogaster*) were infected orally in fly feeding assay in which flies were anesthetized, starved of food and water for several hours, and separated into vials containing filter paper disks inoculated with freshly grown *P. aeruginosa* PA14, as well as 5% sucrose agar (sterile) with and without 50 µg mL^−1^ cPAC alone or in combination with 256 µg mL^−1^ SMX. Post infection mortality of flies was monitored daily for 14 days, with each treatment tested twice in triplicate. Additional details can be found in the Supporting Information.

*In Vivo Infection Assay Using Galleria Mellonella Larvae*: Twenty *G. mellonella* larvae were injected with active cultures of *P. aeruginosa* per treatment. All injected larvae were incubated in Petri dishes at 28 °C under 30% relative humidity in the dark, and the number of dead larvae was scored daily post infection. A larva was considered dead when it displayed no vital signs in response to touch, followed by increased melanization. Additional details are given in the Supporting Information.

*Biofilm Assays*: Biofilm formation was quantified using the standard microtiter plate model^60^ and crystal violet staining, the details of which are described in Appendix S1 (Supporting Information). For biofilm imaging and analysis, biofilms were grown for 16 h at 37 °C under static conditions, after which planktonic cells were removed by rinsing. The cell‐membrane impermeable fluorescent stain TOTO‐1 (Thermo Fisher) and membrane permeable stain SYTO 60 (Thermo Fisher) were added to final working concentrations of 2 × 10^−6^ and 10 × 10^−6^ m, respectively.^61^ Biofilms were imaged as *z*‐stacks using a 63× objective on a Zeiss 800 confocal laser scanning microscope and rendered such that living cells are colored green, and dead cells are colored red as described in the Supporting Information.

*Modified Disk‐Diffusion Assay*: A modified disk‐diffusion assay^62^ was used to detect recovery of antibiotic‐exposed cells. This modified disk‐diffusion assay was conducted in two steps, where Step 1 was similar to classical disk‐diffusion assay and Step 2 was slightly modified. Briefly, an overnight bacterial culture (MHB‐II broth, 37 °C, 200 rpm) was diluted into fresh MHB‐II broth to 10^6^ CFU mL^−1^ and plated on MHB‐II agar plate. Step 1: commercially available TET 30 µg or MIN 30 µg antibiotic disk (Thermo Scientific Oxoid, Fisher Scientific, Canada) was placed on top of the inoculated agar surface and the plate was incubated at 37 °C for 18 h. Custom disks were prepared using sterile blank disks (Thermo Scientific Oxoid, Fisher Scientific, Canada) supplemented with 400 µg glucose or 400 µg cPAC with or without 400 µg glucose and left to dry at room temperature. Step 2: The antibiotic disk was carefully replaced with custom disks containing glucose or cPAC or their combination, and the plate was incubated at 37 °C for an additional 18 h.

*Emergence of Resistance Analysis*: For characterization of in vitro resistance evolution by standard sequential passaging technique,^63^, ^64^ *E. coli* CFT073 and *P. aeruginosa* PA14 cells were grown to exponential phase in MHB‐II at 37 °C. MICs were determined by preparing twofold serial dilutions in 96‐well microtiter plates. Each well was inoculated with the desired bacterial strain, and the plate was incubated at 37 °C for 18 h under static conditions. Bacterial growth was assessed by i) monitoring the cell growth (observed as a pellet and turbidity) in the wells^58^ and ii) monitoring the optical density of the cell suspension in each well at 600 nm (OD_600nm_). Bacterial suspension from sub‐MIC (0.5 × MIC) of TET or cPAC or cPAC+TET combination was used to prepare the inoculum for the next day MIC experiment by diluting it to a final concentration of ≈10^6^ CFU mL^−1^ in MHB‐II. This was repeated for 21 passages, and the ratio of the MIC obtained during each day relative to the MIC at 0 day (first time exposure) was determined. The data were expressed as relative fold increase in MIC with each day or passage. Experiments were performed with biological replicates.

*Membrane Permeabilization and Membrane Integrity Assays*: The outer membrane permeabilization activity of cPAC was determined by the NPN (Sigma‐Aldrich, Canada) assay,^65^ with some modifications. Overnight bacterial cultures were diluted 1:1 in MHB‐II medium to a final volume of 10 mL, with or without supplementation of cPAC or GEN (positive control), and grown to an OD_600_ of 0.5–0.6 (37 °C, 200 rpm). The cells were harvested, washed, resuspended 4‐(2‐hydroxyethyl)‐1‐piperazineethanesulfonic acid (HEPES) buffer containing 1 × 10^−3^ m *N*‐ethylmaleimide (NEM; Sigma‐Aldrich, Canada). Aliquots were mixed with NPN to a final concentration of 10 × 10^−6^ m (in cell suspension), and fluorescence was measured using the microplate reader (excitation: 350 nm; emission: 420 nm). The BacLight kit (L‐13152; Invitrogen, Life Technologies Inc., Canada) was used to assess cell membrane damage,^66^ with fluorescence readings from samples normalized to the values obtained from the untreated control to determine the ratio of membrane‐compromised cells to cells with intact membranes. Details can be found in the Supporting Information.

*EtBr Efflux Assay*: To assess the effect of cPAC on the inhibition of the proton motive force–driven multidrug efflux pump, an EtBr efflux assay was performed.^67^ An overnight‐grown culture of each strain was diluted 1:100 in MHB‐II broth to a final volume of 10 mL and was grown to an OD_600_ of 0.8–1.0 (at 37 °C, 150 rpm). Cells were loaded in polystyrene microcentrifuge tubes (2 mL) and mixed with 5 × 10^−6^ m EtBr and cPACs at 25% of their MIC or a 100 × 10^−6^ m concentration of the proton conductor CCCP (Sigma‐Aldrich Canada) as a positive control. Replica tubes that did not receive cPACs or proton conductor served as negative controls. The tubes were incubated for 1 h (37 °C, 150 rpm). The inoculum then was adjusted to an OD_600_ of 0.4 with MHB‐II broth containing 5 × 10^−6^ m EtBr, and 2 mL of aliquot of this mixture was pelleted (5000 × *g*, 10 min, 4 °C). The pellets were incubated on ice immediately, resuspended in 1 mL of MHB‐II, and aliquoted (200 µL) into a polystyrene 96‐well, black, clear‐bottom plate (Corning, Fisher Scientific Canada). EtBr efflux from the cells was monitored at room temperature using the microplate reader (excitation wavelength 530 nm; emission wavelength, 600 nm). Readings were taken at 5 min intervals for 1 h to monitor efflux pump activity. The background fluorescence of the medium was subtracted from all measurements, and the assay was repeated independently in triplicate.

*In Silico Docking Analysis*: 3DLigandSite server was used to predict ligand binding sites on targeted protein structures using an automated method by searching a structural library to identify homologous structures with bound ligands.^68^ These bound ligands were superimposed onto the targeted protein structure to predict a ligand‐binding site. In silico docking was performed using the Autodock Vina tool without the incorporation of water molecules.^69^ In silico docking analysis by Autodock Vina was compared with SwissDock web service (http://www.swissdock.ch/) to confirm the accuracy and robustness of predicted docking complexes. The ligand docking methods are described in the Supporting Information.

*Statistical Analysis*: Where indicated, a two‐tailed Student's *t*‐test (*P* < 0.05) was used to determine whether the presence of cPAC resulted in a significant difference compared to levels for the control. A two‐way analysis of variance (ANOVA), followed by Sidak's multiple comparison, was used for biofilm assays to analyze statistical significance of the difference in biomass. Fruit fly survival curves were prepared using GraphPad Prism 6 (GraphPad Software, Inc., San Diego, CA) to perform a statistical log–rank (Mantel–Cox) test. Throughout the text, all of the changes (increase or decrease) reported were statistically significant. Statistically nonsignificant values were not mentioned in the text.

*Full Methods*: Detailed procedures of all methods are available in the Supporting Information.

## Conflict of Interest

Authors Nathalie Tufenkji and Vimal B. Maisuria have applied for a patent (WO 2017/096484) on the use of cranberry derived phenolic compounds as antibiotic synergizing agent against pathogenic bacteria. The patent application is presently under review by the USPTO, with both authors listed as inventors. All authors declare that they have no other conflicts of interest.

## Supporting information

Supplementary

## References

[1] J. W. Costerton , P. S. Stewart , E. P. Greenberg , Science 1999, 284, 1318. 10334980 10.1126/science.284.5418.1318

[2] J. C. Kester , S. M. Fortune , Crit. Rev. Biochem. Mol. Biol. 2014, 49, 91. 24328927 10.3109/10409238.2013.869543

[3] K. Lewis , Nat. Rev. Microbiol. 2007, 5, 48. 17143318 10.1038/nrmicro1557

[4] D. Davies , Nat. Rev. Drug Discovery 2003, 2, 114. 12563302 10.1038/nrd1008

[5] R. A. Fisher , B. Gollan , S. Helaine , Nat. Rev. Microbiol. 2017, 15, 453. 28529326 10.1038/nrmicro.2017.42

[6] N. R. Blatherwick , Arch. Intern. Med. 1914, 14, 409.

[7] J. K. Crellin , J. Philpott , A. L. T. Bass , A Reference Guide to Medicinal Plants, Duke University Press, Durham, NC, USA 1990.

[8] I. A. Eydelnant , N. Tufenkji , Langmuir 2008, 24, 10273. 18698853 10.1021/la801525d

[9] N. Tufenkji , O. J. Rifai , K. Harmidy , I. A. Eydelnant , Food Res. Int. 2010, 43, 922.

[10] K. Gupta , M. Y. Chou , A. Howell , C. Wobbe , R. Grady , A. E. Stapleton , J. Urol. 2007, 177, 2357. 17509358 10.1016/j.juro.2007.01.114 PMC3684265

[11] A. B. Howell , J. D. Reed , C. G. Krueger , R. Winterbottom , D. G. Cunningham , M. Leahy , Phytochemistry 2005, 66, 2281. 16055161 10.1016/j.phytochem.2005.05.022

[12] M. Chan , G. Hidalgo , B. Asadishad , S. Almeida , N. Muja , M. S. Mohammadi , S. N. Nazhat , N. Tufenkji , Colloids Surf., B 2013, 110, 275. 10.1016/j.colsurfb.2013.03.047 23732805

[13] G. Hidalgo , M. Chan , N. Tufenkji , Appl. Environ. Microbiol. 2011, 77, 6852. 21821749 10.1128/AEM.05561-11 PMC3187111

[14] J. McCall , G. Hidalgo , B. Asadishad , N. Tufenkji , Can. J. Microbiol. 2013, 59, 430. 23750959 10.1139/cjm-2012-0744

[15] C. O'May , A. Ciobanu , H. Lam , N. Tufenkji , Biofouling 2012, 28, 1063. 23020753 10.1080/08927014.2012.725130

[16] C. O'May , N. Tufenkji , Appl. Environ. Microbiol. 2011, 77, 3061. 21378043 10.1128/AEM.02677-10 PMC3126419

[17] R. K. Ulrey , S. M. Barksdale , W. Zhou , M. L. van Hoek , BMC Complementary Altern. Med. 2014, 14, 499. 10.1186/1472-6882-14-499 PMC4320558 25511463

[18] G. Hidalgo , A. Ponton , J. Fatisson , C. O'May , B. Asadishad , T. Schinner , N. Tufenkji , Appl. Environ. Microbiol. 2011, 77, 1532. 21169441 10.1128/AEM.02201-10 PMC3067223

[19] V. B. Maisuria , Y. L. Los Santos , N. Tufenkji , E. Deziel , Sci. Rep. 2016, 6, 30169. 27503003 10.1038/srep30169 PMC4977528

[20] P. Di Martino , R. Agniel , K. David , C. Templer , J. L. Gaillard , P. Denys , H. Botto , World J. Urol. 2006, 24, 21. 16397814 10.1007/s00345-005-0045-z

[21] R. G. Jepson , G. Williams , J. C. Craig , Cochrane Database Syst. Rev. 2012, 10, Cd001321. 23076891 10.1002/14651858.CD001321.pub5 PMC7027998

[22] J. P. Lavigne , G. Bourg , C. Combescure , H. Botto , A. Sotto , Clin. Microbiol. Infect. 2008, 14, 350. 18190583 10.1111/j.1469-0691.2007.01917.x PMC4749672

[23] Y. Liu , M. A. Black , L. Caron , T. A. Camesano , Biotechnol. Bioeng. 2006, 93, 297. 16142789 10.1002/bit.20675

[24] R. Raz , B. Chazan , M. Dan , Clin. Infect. Dis. 2004, 38, 1413. 15156480 10.1086/386328

[25] A. B. Howell , Mol. Nutr. Food Res. 2007, 51, 732. 17487930 10.1002/mnfr.200700038

[26] M. S. Diarra , G. Block , H. Rempel , B. D. Oomah , J. Harrison , J. McCallum , S. Boulanger , É. Brouillette , M. Gattuso , F. Malouin , BMC Complementary Altern. Med. 2013, 13, 90. 10.1186/1472-6882-13-90 PMC3641957 23622254

[27] The Selection and Use of Essential Medicines, Report of the WHO Expert Committee (including the 20th WHO Model List of Essential Medicines and the 6th WHO Model List of Essential Medicines for Children), WHO technical report series; report no. 1006, World Health Organization, Geneva 2017, pp. 62–172.

[28] L. Ejim , M. A. Farha , S. B. Falconer , J. Wildenhain , B. K. Coombes , M. Tyers , E. D. Brown , G. D. Wright , Nat. Chem. Biol. 2011, 7, 348. 21516114 10.1038/nchembio.559

[29] L. Pumbwe , L. J. Piddock , Antimicrob. Agents Chemother. 2000, 44, 2861. 10991874 10.1128/aac.44.10.2861-2864.2000 PMC90165

[30] T. Eicher , M. A. Seeger , C. Anselmi , W. Zhou , L. Brandstätter , F. Verrey , K. Diederichs , J. D. Faraldo‐Gómez , K. M. Pos , Elife 2014, 3, 10.7554/eLife.03145.002. PMC4359379 25248080

[31] Y. Morita , J. Tomida , Y. Kawamura , Front. Microbiol. 2014, 4, 422. 24409175 10.3389/fmicb.2013.00422 PMC3884212

[32] B. Lesic , F. Lépine , E. Déziel , J. Zhang , Q. Zhang , K. Padfield , M. H. Castonguay , S. Milot , S. Stachel , A. A. Tzika , R. G. Tompkins , L. G. Rahme , PLoS Pathog. 2007, 3, 1229. 17941706 10.1371/journal.ppat.0030126 PMC2323289

[33] T. R. Shryock , Nat. Rev. Microbiol. 2004, 2, 425. 15100695 10.1038/nrmicro887

[34] D. A. Rasko , V. Sperandio , Nat. Rev. Drug Discovery 2010, 9, 117. 20081869 10.1038/nrd3013

[35] M. Rhen , S. Eriksson , M. Clements , S. Bergstrom , S. J. Normark , Trends Microbiol. 2003, 11, 80. 12598130 10.1016/s0966-842x(02)00038-0

[36] S. S. Grant , D. T. Hung , Virulence 2013, 4, 273. 23563389 10.4161/viru.23987 PMC3710330

[37] A. Vadekeetil , V. Alexandar , S. Chhibber , K. Harjai , Microb. Pathog. 2016, 90, 98. 26620081 10.1016/j.micpath.2015.11.024

[38] C. M. O'Hara , F. W. Brenner , J. M. Miller , Clin. Microbiol. Rev. 2000 13, 534. 11023955 10.1128/cmr.13.4.534-546.2000 PMC88947

[39] M. Bassetti , A. Vena , A. Croxatto , E. Righi , B. Guery , Drugs Context 2018, 7, 212527. 29872449 10.7573/dic.212527 PMC5978525

[40] J. M. Blair , M. A. Webber , A. J. Baylay , D. O. Ogbolu , L. J. Piddock , Nat. Rev. Microbiol. 2015, 13, 42. 25435309 10.1038/nrmicro3380

[41] G. Cox , G. D. Wright , Int. J. Med. Microbiol. 2013, 303, 287. 23499305 10.1016/j.ijmm.2013.02.009

[42] D. Du , Z. Wang , N. R. James , J. E. Voss , E. Klimont , T. Ohene‐Agyei , H. Venter , W. Chiu , B. F. Luisi , Nature 2014, 509, 512. 24747401 10.1038/nature13205 PMC4361902

[43] Z. Wang , G. Fan , C. F. Hryc , J. N. Blaza , I. I. Serysheva , M. F. Schmid , W. Chiu , B. F. Luisi , D. Du , Elife 2017, 6, 10.7554/eLife.24905.001. PMC5404916 28355133

[44] R. Nakashima , K. Sakurai , S. Yamasaki , K. Hayashi , C. Nagata , K. Hoshino , Y. Onodera , K. Nishino , A. Yamaguchi , Nature 2013, 500, 102. 23812586 10.1038/nature12300

[45] L. Y. Foo , Y. Lu , A. B. Howell , N. Vorsa , Phytochemistry 2000, 54, 173. 10872208 10.1016/s0031-9422(99)00573-7

[46] A. V. Vargiu , P. Ruggerone , T. J. Opperman , S. T. Nguyen , H. Nikaido , Antimicrob. Agents Chemother. 2014, 58, 6224. 25114133 10.1128/AAC.03283-14 PMC4187987

[47] M. Kvist , V. Hancock , P. Klemm , Appl. Environ. Microbiol. 2008, 74, 7376. 18836028 10.1128/AEM.01310-08 PMC2592912

[48] L. J. Piddock , Nat. Rev. Microbiol. 2006, 4, 629. 16845433 10.1038/nrmicro1464

[49] J. Glavis‐Bloom , M. Muhammed , E. Mylonakis , Adv. Exp. Med. Biol. 2012, 710, 11. 22127881 10.1007/978-1-4419-5638-5_2

[50] D. O'Callaghan , A. Vergunst , Curr. Opin. Microbiol. 2010, 13, 79. 20045373 10.1016/j.mib.2009.12.005

[51] S. Tanabe , J. Santos , V. D. La , A. B. Howell , D. Grenier , Molecules 2011, 16, 2365. 21399573 10.3390/molecules16032365 PMC6259657

[52] R. Rajbhandari , N. Peng , R. Moore , A. Arabshahi , J. M. Wyss , S. Barnes , J. K. Prasain , J. Agric. Food Chem. 2011, 59, 6682. 21634376 10.1021/jf200673h PMC3165050

[53] H. L. Mobley , J. W. Warren , J. Clin. Microbiol. 1987, 25, 2216. 3320089 10.1128/jcm.25.11.2216-2217.1987 PMC269446

[54] L. G. Rahme , E. J. Stevens , S. F. Wolfort , J. Shao , R. G. Tompkins , F. M. Ausubel , Science 1995, 268, 1899. 7604262 10.1126/science.7604262

[55] N. T. Liberati , J. M. Urbach , S. Miyata , D. G. Lee , E. Drenkard , G. Wu , J. Villanueva , T. Wei , F. M. Ausubel , Proc. Natl. Acad. Sci. USA 2006, 103, 2833. 16477005 10.1073/pnas.0511100103 PMC1413827

[56] M. G. Lamarche , E. Déziel , PLoS One 2011, 6, e24310. 21957445 10.1371/journal.pone.0024310 PMC3177830

[57] M. A. Martín , S. Ramos , R. Mateos , J. P. J. Marais , L. Bravo‐Clemente , C. Khoo , L. Goya , Food Res. Int. 2015, 71, 68.

[58] Clinical and Laboratory Standards Institute Methods for Dilution Antimicrobial Susceptibility Tests for Bacteria That Grow Aerobically, CLSI document M07‐A8, Approved Standard, 7th ed., Clinical and Laboratory Standards Institute, Wayne, PA 2009, pp. 18–20.

[59] J. C. Palomino , A. Martin , M. Camacho , H. Guerra , J. Swings , F. Portaels , Antimicrob. Agents Chemother. 2002, 46, 2720. 12121966 10.1128/AAC.46.8.2720-2722.2002 PMC127336

[60] J. Rosenblatt , R. Reitzel , T. Dvorak , Y. Jiang , R. Y. Hachem , I. I. Raad , Antimicrob. Agents Chemother. 2013, 57, 3555. 23070163 10.1128/AAC.01287-12 PMC3535935

[61] M. Okshevsky , R. L. Meyer , J. Microbiol. Methods 2014, 105, 102. 25017901 10.1016/j.mimet.2014.07.010

[62] O. Gefen , B. Chekol , J. Strahilevitz , N. Q. Balaban , Sci. Rep. 2017, 7, 41284. 28145464 10.1038/srep41284 PMC5286521

[63] X. Yang , S. Goswami , B. K. Gorityala , R. Domalaon , Y. Lyu , A. Kumar , G. G. Zhanel , F. Schweizer , J. Med. Chem. 2017, 60, 3913. 28399372 10.1021/acs.jmedchem.7b00156

[64] L. L. Ling , T. Schneider , A. J. Peoples , A. L. Spoering , I. Engels , B. P. Conlon , A. Mueller , T. F. Schäberle , D. E. Hughes , S. Epstein , M. Jones , L. Lazarides , V. A. Steadman , D. R. Cohen , C. R. Felix , K. A. Fetterman , W. P. Millett , A. G. Nitti , A. M. Zullo , C. Chen , K. Lewis , Nature 2015, 517, 455. 25561178 10.1038/nature14098 PMC7414797

[65] T. J. Falla , D. N. Karunaratne , R. E. Hancock , J. Biol. Chem. 1996, 271, 19298. 8702613 10.1074/jbc.271.32.19298

[66] L. Boulos , M. Prevost , B. Barbeau , J. Coallier , R. Desjardins , J. Microbiol. Methods 1999, 37, 77. 10395466 10.1016/s0167-7012(99)00048-2

[67] V. B. Maisuria , Z. Hosseinidoust , N. Tufenkji , Appl. Environ. Microbiol. 2015, 81, 3782. 25819960 10.1128/AEM.00239-15 PMC4421064

[68] M. N. Wass , L. A. Kelley , M. J. Sternberg , Nucleic Acids Res. 2010, 38, W469. 20513649 10.1093/nar/gkq406 PMC2896164

[69] O. Trott , A. J. Olson , J. Comput. Chem. 2010, 31, 455. 19499576 10.1002/jcc.21334 PMC3041641

