# Proanthocyanidin Interferes with Intrinsic Antibiotic Resistance Mechanisms of Gram‐Negative Bacteria

**DOI:** 10.1002/advs.202202641

**Published:** 2022-07-15

**Authors:** Vimal B. Maisuria, Mira Okshevsky, Eric Déziel, Nathalie Tufenkji

*Adv. Sci*. **2019**, 1802333

DOI: 10.1002/advs.201802333

## 1 Introduction

The authors wish to correct Figures 1 and 5G, as well as Supporting Figures S1a and S3 of the original manuscript, in which some errors were detected in the FICI values due to mistakes in normalization of the resazurin assay (fluorescence) measurements in the checkerboard experiments. The corrected figures (Figures 1 and 5G, Supporting Figures S1a and S3) are presented below along with updated text for the associated Results section. The overall conclusions of the study remain unchanged.

## 2 Results

### 2.1 cPAC Potentiates a Broad Range of Antibiotics In Vitro

(…)

**Figure** **1**A–C show positive and/or negative interactions between cPAC and antibiotics against bacterial pathogens. FICI values ≤ 0.5 (indicated by the gray zone) demonstrate that cPAC potentiated the effectiveness of nitrofurantoin (NIT), kanamycin (KAN), tetracycline (TET), and azithromycin (AZT) to inhibit the growth of *E. coli* CFT073 and *P. aeruginosa* PA14 using up to 94% less antibiotic than that required in the absence of cPAC. cPAC also potentiated gentamicin (GEN) and fosfomycin (FOS) activity to inhibit the growth of *P. mirabilis* HI4320; up to 75% less antibiotic was needed than in the absence of cPAC. In the case of *P. aeruginosa* strain PAO1, cPAC enhanced the efficiency of the antibiotics SMX, FOS, GEN, and AZT (Figure S1A, Supporting Information). It is worth noting that several combinations of cPAC and antibiotic resulted in FICI values between 0.5 – 1 which also led to a decrease in MIC of the antibiotic (e.g., SMX for *E. coli* CFT073, TET for *P. mirabilis* HI4320).

**Figure 1:**
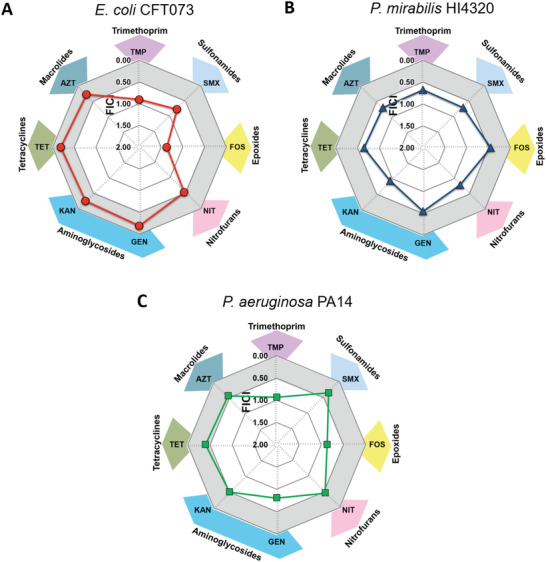
Potentiating interaction of cPAC with antibiotic results in growth inhibition. MICs were determined for the combination of cPAC with each antibiotic in vitro. Fractional inhibitory concentration index (FICI) for each combination are shown for (A) *E. coli* CFT073, (B) *P. mirabilis* HI4320, and (C) *P. aeruginosa* PA14. A FICI ≤0.5 is indicated by the gray shaded area. TMP: trimethoprim; SMX: sulfamethoxazole; FOS: fosfomycin; NIT: nitrofurantoin; GEN: gentamicin; KAN: kanamycin; TET: tetracycline; AZT: azithromycin.

(…)

We investigated the interaction of cPAC with co‐trimoxazole [the combination of SMX and TMP, commonly used to treat urinary tract infections and bacterial dysentery^[27]^]. cPAC enhanced the synergistic efficacy of co‐trimoxazole, reducing the MIC up to 4‐fold against *P. mirabilis* HI4320. In the case of *P. aeruginosa* PA14, combination of cPAC with co‐trimoxazole decreased the MIC by 2‐fold, which is more effective than the potentiating combination of cPAC with TMP alone (Figure S3, Supporting Information). (…)

### 2.2 Mechanisms by Which cPAC Potentiates Antibiotic Activity

(…)

The mutant strain with three nonfunctioning efflux pumps was more susceptible to TET compared to the wild‐type strain, such that addition of cPAC provided no further benefit for the potency of TET. Therefore, potentiation between cPAC and TET was not observed in the efflux pump‐nonfunctioning mutant (*oprM‐*) (**Figure** **5**G). (…)

**Figure 5:**
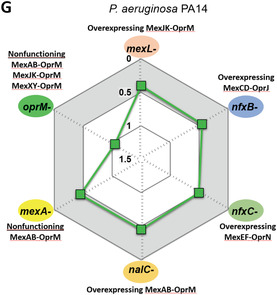
Mechanisms of antibiotic potentiation by cPAC. (…) (G) MICs were determined for the combination of cPAC with TET in vitro. FICI values for cPAC+TET combination are shown for efflux pump mutants of *P. aeruginosa* PA14: *mexL^−^* (overexpressing MexJK‐OprM), *nfxB^−^* (overexpressing MexCD‐OprJ), *nfxC^−^* (overexpressing MexEF‐OprN), *nalC^−^* (overexpressing MexAB‐OprM), *mexA^−^* (nonfunctioning MexAB‐OprM), and *oprM^−^* (nonfunctioning MexAB‐OprM, MexJK‐OprM and MexXY‐OprM). A FICI index ≤0.5 is indicated by the gray shaded area.

## Supporting information

Supporting Information

