# Supplementary material for: Proanthocyanidin Interferes with Intrinsic Antibiotic Resistance Mechanisms of Gram‐Negative Bacteria
Source: Adv Sci (Weinh). 2019 May 28;6(15):1802333. doi: 10.1002/advs.201802333 (PMC6685479; doi:10.1002/advs.201802333)
Supplement: Supplementary file 1 — Supplementary [file ADVS-6-1802333-s001.pdf]

Copyright WILEY-VCH Verlag GmbH & Co. KGaA, 69469 Weinheim, Germany, 2019.

ADVANCED SCIENCE
Open Access

Supporting Information

for *Adv. Sci.,* DOI: 10.1002/advs.201802333

Proanthocyanidin Interferes with Intrinsic Antibiotic Resistance Mechanisms of Gram-Negative Bacteria

*Vimal B. Maisuria, Mira Okshevsky, Eric Déziel, and Nathalie Tufenkji**

Supporting Information

Proanthocyanidin interferes with intrinsic antibiotic resistance mechanisms of Gram-negative bacteria

Vimal B. Maisuria, Mira Okshevsky, Eric Déziel, Nathalie Tufenkji

Corresponding author: Nathalie Tufenkji

E-mail: nathalie.tufenkji@mcgill.ca

SI Experimental Procedures

Determination of MICs

In the resazurin microtiter plate assay, each well of a microtiter plate was supplemented with 100 μM resazurin and incubated in the dark for 20 min at room temperature, followed by fluorescence measurements at excitation and emission wavelengths of 570 and 590 nm, respectively, using a Tecan Infinite M200 Pro microplate reader (Tecan Group Ltd., Switzerland). The concentrations of cPAC used in this study (200, 100, 50, 25 and 12.5 µg/mL) correspond to molar concentrations of 347, 173.5, 86.8, 43.4 and 21.7 µM, respectively. This conversion is based on the molecular weight of the predominant structure, the PAC-A2 dimer. The lowest concentration of a compound able to prevent an increase in the growth, OD_{600} and an increase in resazurin fluorescence intensity was recorded as the MIC for that compound.[1]

Checkerboard microdilution assay

The checkerboard microdilution assay[2] was used for evaluation of *in vitro* antimicrobial synergy between two compounds (i.e., antibiotic/cPAC). Twofold serial dilutions were prepared in MHB-II for each of the two compounds under study. The serial dilutions then were loaded into 96-well plates to achieve combinations having different concentrations of each of the two compounds. Each well subsequently was inoculated with approximately 10^6 CFU ml^{-1} of the desired bacterial strain and incubated at 37°C for 18 h under static conditions. The fractional inhibitory concentration index (FICI) for each combination was calculated by using the following formulas[2] : $FIC_{component\ 1} = MIC_{component\ 1,\ in\ combination}/MIC_{component\ 1,\ alone}$ and $FICI = FIC_{component\ 1} + FIC_{component\ 2}$. An FICI of ≤0.5 indicated synergy, an FICI of >0.5 and ≤4 indicated no interaction/indifference, and an FICI of >4 indicated antagonism.[2]

In vivo infection assay using *Drosophila melanogaster* flies

Fruit flies (*D. melanogaster*) were infected orally in fly feeding assay as before[3,4], with some modifications. Briefly, flies were anesthetized under a gentle stream of carbon dioxide. Male

flies (3- to 5-days-old) were starved of food and water for 5–6 h and separated into vials (10 per vial) containing 5 mL of 5% sucrose agar (sterile) without and with 50 μg mL^{-1} cPAC alone or in combination with 256 μg mL^{-1} SMX and 2.3-cm filter paper disks containing freshly grown bacterial culture suspension of *P. aeruginosa* PA14. To achieve this freshly grown culture, an overnight PA14 culture was inoculated in 6 mL Tryptic Soy broth medium (BD Bacto, Fisher Scientific Canada) and incubated at 37°C and 100 rpm until $OD_{600} = 3.0$. This culture was centrifuged at 12,000 × *g* for 1 min and the resulting pellet resuspended in 150 μL sterile 5% sucrose, without and with 50 μg mL^{-1} cPAC or 256 μg mL^{-1} SMX. All filters were soaked appropriately with this culture suspension, along with sucrose agar, in feeding vials prior to transferring flies into the vial. Separate feeding vials soaked with 150 μL of 5% sucrose without and with 50 μg mL^{-1} cPAC or 256 μg mL^{-1} SMX were used as negative or uninfected controls for each experiment. Post-infection mortality of flies was monitored daily for 14 days, with each treatment tested twice in triplicate.

***In vivo* infection assay using *Galleria mellonella* larvae**

Galleria mellonella infection assay was performed as described previously with some modification[5]. Briefly, *P. aeruginosa* PA14 culture grown overnight in TSB broth were diluted 1:100 in the same growth medium and further grown to OD_{600} of 1.17 at 37°C under 100 rpm shaking. The freshly grown culture was centrifuged, and pellets were washed and resuspended in 10 mM $MgSO_4$ to an OD_{600} of 0.1. To prepare lethal dose of *P. aeruginosa* PA14 culture serial 10-fold dilutions were made in 10 mM $MgSO_4{\cdot}7H_2O$ supplemented with 1 mg mL^{-1} ampicillin (adjusted to 30 ± 5cells in 5 µL of suspension). Ampicillin was used to prevent infection by bacterial contaminants on the surface of the larvae at a final concentration of approximately 5 ng per larva during survival phase. The treatment dose of 50 μg mL^{-1} cPAC alone or in combination with 256 μg mL^{-1} SMX prepared in 10 mM $MgSO_4{\cdot}7H_2O$. Twenty larvae were injected per each treatment and assay was performed twice. cPAC at 50 μg mL^{-1} and SMX at 256 μg mL^{-1} were assessed individually to monitor the adverse effect on larvae survival. To analyze effect of cPAC at 50 μg mL^{-1} on *G. mellonella* survival post-PA14 infection, additional set of larvae were first injected with 5 µL aliquot of lethal bacterial dose at their proleg, and after 3 h they were injected at second proleg with 5 µL saline solution containing or not cPAC without and with SMX using prewashed and surface sterilized Hamilton syringe. All injected larvae were incubated in Petri

dishes at 28°C under 30% relative humidity in the dark, and the number of dead larvae was scored daily post infection. A larva was considered dead when it displayed no vital signs in response to touch, followed by increased melanization. A mock inoculation using prewashed Hamilton syringe was also performed in each experiment to monitor the killing due to physical injury or infection by pathogenic contaminants. The timing of the treatment (3 hr after infection) was based on LD_{50} and time course of *P. aeruginosa*PA14 infection in *Galleria* larvae as described in the literature[5,6]. Colonization levels were not monitored in the infection models.

Biofilm assays

Biofilm formation was quantified using the standard microtiter plate model.[7] Briefly, overnight cultures (MHB-II broth, 37°C, 200 rpm) were diluted into fresh MHB-II broth (with or without cPAC and/or SMX) to 10^6 CFU mL^{-1}. A range of concentrations of the SMX (64 to 1024 μg mL^{-1}) and two concentrations of cPAC (50 and 100 μg mL^{-1}) was chosen against all four bacterial strains. Concentrations were achieved using 2-fold serial dilutions as mentioned in above section of MIC determination assay. Aliquots (100 μL) of these cultures were transferred into the wells of polystyrene, flat-bottom, untreated 96-well plates (Falcon, Corning) in triplicate. For all assays, biofilms were allowed to develop for 16 h at 37°C under static conditions, after which OD_{600} values were recorded, the spent broth was decanted from the wells, and the wells were gently rinsed three times with sterile 10 mM phosphate-buffered saline (PBS, pH 7.2). The washed biofilm was stained with crystal violet (CV). For the CV stain assay, 100 μl of 0.1% (w/v) CV was loaded in each well, and the plates were incubated for 15 min under static conditions at room temperature. The wells subsequently were rinsed with DI water to remove excess dye, and the CV adsorbed to the biomass in each well was solubilized in 100 μl of absolute ethanol for 10 min. The solubilized CV was quantified (at OD_{570}) using a Tecan Infinite M200 Pro microplate reader (Tecan Group Ltd., Switzerland). Control experiments were performed with cell-free broth to adjust for background signal. For biofilm imaging and analysis, preculture of *P. aeruginosa* PA14 was prepared as above and diluted into fresh MHB-II broth (with or without cPACs and/or SMX) to 10^6 CFU mL^{-1}, and 100 μL dispensed into the wells of an uncoated 96-well μ-plates (ibidi, Germany). Biofilms were incubated 16 h at 37°C under static conditions, after which OD_{600} values were recorded, the spent broth was decanted from the wells, and the wells were gently rinsed six times with 10 mM PBS (pH 7.2) to remove planktonic

cells. The cell-membrane impermeable fluorescent stain TOTO-1 (Thermo Fisher) and membrane permeable stain SYTO 60 (Thermo Fisher) were added to a final working concentration of 2 µM and 10 µM, respectively.[8] Biofilms were imaged as z-stacks using a 63× objective on a Zeiss 800 confocal laser scanning microscope. Imaris image analysis software v. 8.3 (bitplane) was used to quantify biomass. Biomass surfaces were rendered using a constant threshold value for all treatments, and total biomass volume was calculated for red (SYTO 60) and green (TOTO-1) channels separately. Zen lite software v. 2.3 (Zeiss) was used to render 3D images in transparency mode. The colors of the red channel (SYTO 60) and green channel (TOTO-1) were switched to facilitate intuitive image interpretation, so that living cells are colored green, and dead cells as well as extracellular biomass debris are colored red.

Bacterial growth assays

Growth assays were performed in MHB-II broth cultures and set up as per above assays using 2-fold serial dilution technique. Plates were incubated at 37°C, under shaking (orbital shaking 44.3 rpm) conditions within the microtitre plate reader (Tecan Infinite M200 Pro, Switzerland) and OD_{600} measurements were recorded every 30 min for 18 h.

Membrane integrity assays

The BacLight kit (L-13152; Invitrogen, Life Technologies Inc., Canada) was used to assess cell membrane damage.[9] Overnight bacterial cultures were diluted 1:40 in fresh MHB-II broth to a final volume of 5 ml, grown to an OD_{600} of 0.5 to 0.6, washed with sterile 10 mM PBS (pH 7.0), and resuspended in 1/10 of the original volume. The washed cells then were diluted 1:20 (vol/vol) into 200 µg/mL cPAC or 10 µM CTAB solutions as described in reference[10] or 0.3% (vol/vol) DMSO (control). Cultures were incubated at room temperature (21 ± 2°C) on a tube rocker for 10 min. At the end of the incubation period, an aliquot was taken for CFU counts and the remaining suspension was washed with 10 mM PBS and resuspended to an OD_{600} of 0.3. An aliquot (100 µl) of each bacterial suspension was removed and added to a 96-well, black, clear-bottom plate (Corning, Fisher Scientific Canada) along with an equal volume of the *Bac*Light reagent (2× stock solution, L13152; Invitrogen, Life Technologies Inc., Canada), and the plates were incubated for 10 min at room temperature in the dark. At the end of the incubation period,

fluorescence intensity was recorded for both kit components, SYTO-9 (excitation, 485 nm; emission, 530 nm) and propidium iodide (excitation, 485 nm; emission, 645 nm), using the microplate reader. Fluorescence readings from samples were normalized to the values obtained from the untreated control to determine the ratio of membrane-compromised cells to cells with intact membranes. Cetyltrimethylammonium bromide (CTAB; Sigma-Aldrich Canada), a cationic detergent that is known to cause membrane damage[11], was used at a concentration of 10 μM as a positive control for membrane disruption.

***In silico* docking analysis**

To maintain the search robustness in Autodock Vina tool, ten rounds of iteration modes with three energy range and eight exhaustiveness were used for each docking process.[12] A grid of 20 Å by 20 Å by 20 Å centered in the middle of the predicted pocket was used for ligand docking. *In silico* docking analysis by Autodock Vina was compared with SwissDock web service (http://www.swissdock.ch/) to confirm the accuracy and robustness of predicted docking complexes. The ligand docking under Autodock Vina tool[12] was performed using the Lamarckian genetic algorithm that can identify steric and hydrogen bonding interactions using a semi-empirical free energy force field to evaluate conformations during docking simulations. Rapid energy evaluation was achieved by pre-calculating atomic affinity potentials for each atom type in the ligand molecule being docked. Each of the pair-wise binding energetic term includes evaluations for dispersion/repulsion, hydrogen bonding, electrostatics, and desolvation. SwissDock is a ligand-protein pairwise interactions-based molecular docking tool which identifies binding energies, docking transformations, hydrogen bonding, electrostatics, desolvation and ligand binding modes.[13]The best positions with higher docking interaction energies were sampled and compared in every computed complex. The molecular graphics and ligand volume analyses were performed using the UCSF Chimera 1.10.2. The Computed Atlas of Surface Topography of proteins was used to explore the volumes of the ligand binding cavities in the target proteins (http://sts.bioe.uic.edu/castp/index.php). The established protein complexes of AcrAB-TolC (RCSB PDB 5V5S), AcrB with minocycline (MIN) (RCSB PDB 4U8Y), MexA (RCSB PDB 2V4D), MexB (RCSB PDB 3W9I), OprM (RCSB PDB 3D5K) were used as targets, and A-type cPAC (NCBI PubChem CID 9808627) molecular structure was used as ligands in virtual docking for each protein.

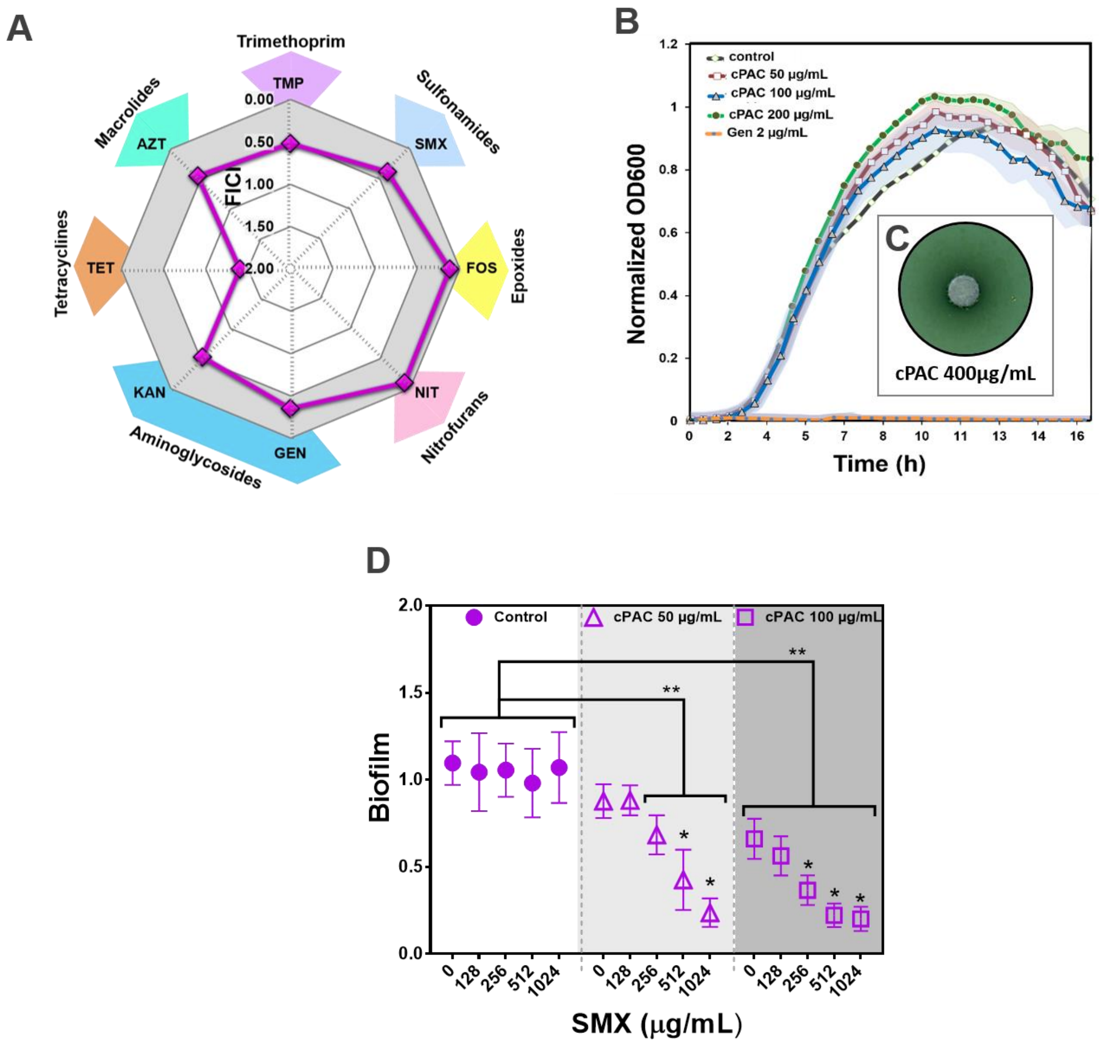

Figure S1. Potentiating action of cPAC with antibiotics for inhibition of growth and biofilm of *P. aeruginosa* PAO1. (**A**)MICs were determined for the combination of cPAC with each antibiotic indicated with their classes *in vitro*. Fractional inhibitory concentration index (FICI) for each combination against *P. aeruginosa*PAO1. FICI of ≤0.5 indicated with gray color at the border. TMP: trimethoprim; SMX: sulfamethoxazole; FOS: fosfomycin; NIT: nitrofurantoin; GEN: gentamicin; KAN: kanamycin; TET: tetracycline; AZT: azithromycin. (**B**) Growth curve of *P. aeruginosa* PAO1 with cPAC or GEN. The graph shows the Normalized OD600 = OD600 – initial OD600 versus time for bacteria grown in MHB-II broth (control) or with cPAC alone (concentration as indicated) or with GEN (MIC 2 µg/mL) alone. Data shown in growth curves are averages of *n*=3 with shaded S.D. (**C**) Growth of *P. aeruginosa* PAO1 on MHB-II agar surface exposed to cPAC disk. A disk containing 400 µg mL^{-1} cPAC was placed on top of the MHB-II agar plated with bacteria and incubated for 24 h. (**D**) Effect of cPAC alone and in combination with SMX on biofilm formation of *P. aeruginosa* PAO1**.** The graph presents normalized biofilm levels (OD_{570nm}/cell OD_{600nm}) versus different sub-inhibitory concentrations of SMX. Statistically significant differences are indicated for each sample treated with cPAC and SMX compared to the control (sample treated with the corresponding concentration of SMX only) (**, *P*<0.01; *, *P*<0.05; Two-way ANOVA) and for samples treated with cPAC plus SMX compared to sample treated with the same concentration of cPAC without SMX (*, *P*<0.05; Two-way ANOVA).

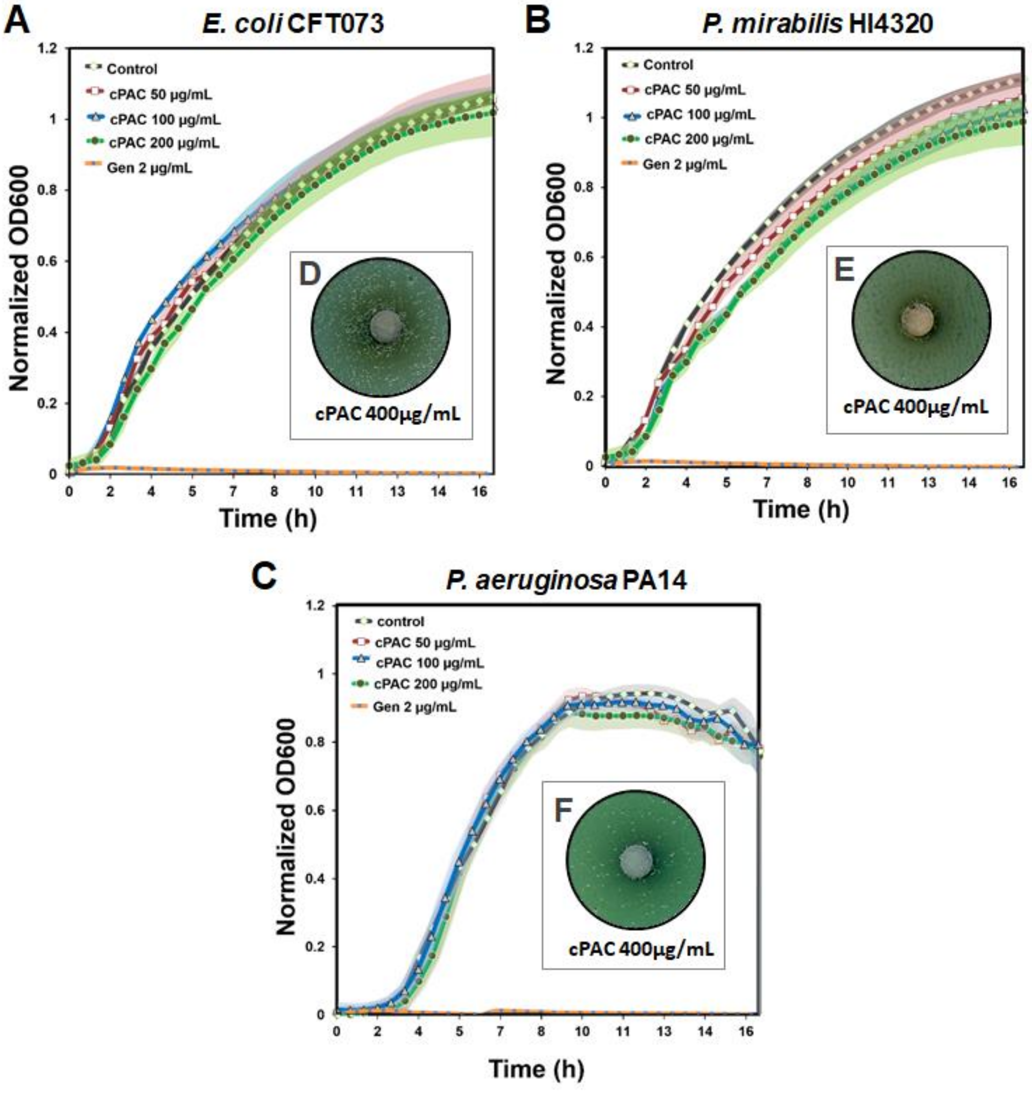

Figure S2. Growth curves for (A) *E. coli* CFT073, (B) *P. mirabilis* HI4320 and (C) *P. aeruginosa* PA14 with cPAC or gentamycin (GEN). The graph shows the Normalized OD600 = OD600 – initial OD600 versus time for bacteria grown in MHB-II broth without cPAC (control) or with cPAC (concentration as indicated) or with GEN (MIC 2 µg/mL). Data shown in growth curves are averages of *n*=3 with shaded S.D. The growth of (**D**) *E. coli* CFT073, (**E**) *P. mirabilis* HI4320 and (**F**) *P. aeruginosa* PA14 on MHB-II agar surface exposed with cPAC disk. The disk containing 400 µg mL^{-1} cPAC was placed on the top of the MHB-II agar plated with bacteria and incubated for 24 h. A bacterial lawn surrounding the disk appeared at the end of the incubation period, indicating no growth inhibition.

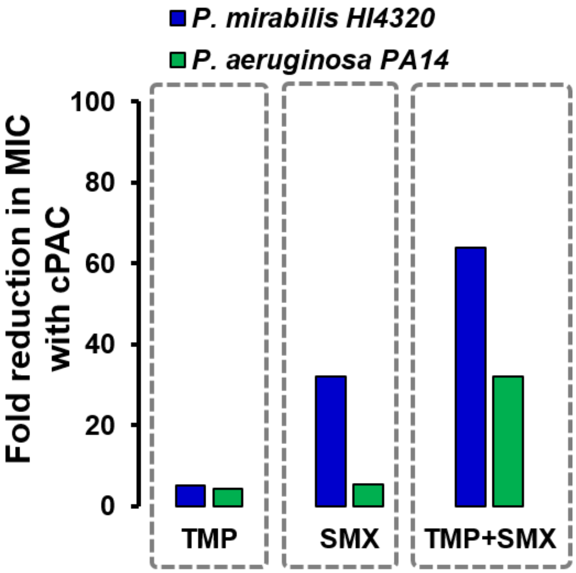

Figure S3. Potentiating interaction of cPAC with antibiotic results in growth inhibition. cPAC reduces the MIC of TMP or SMX alone or in combination (TMP+SMX, co-trimoxazole) for the growth inhibition of *P. mirabilis* HI4320 and *P. aeruginosa* PA14.

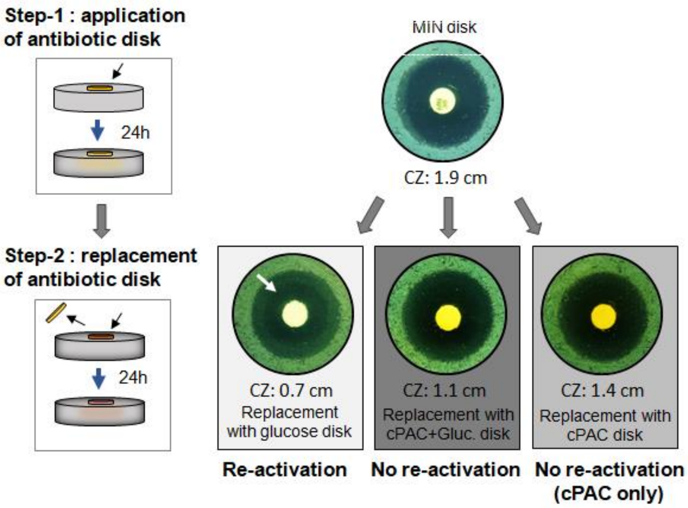

Figure S4. Inhibition of growth re-activation in antibiotic-exposed *E. coli* CFT073. The detection of growth re-activation in bacteria using modified disk-diffusion assay. Step-1: the minocycline (MIN) antibiotic disk was placed on the top of the MHB-II agar and incubated for 24 h, so the antibiotic diffuses from the disk. The thick dashed lines mark the diameter of the clear zone surrounding the disk. Step-2: replacing the MIN disk with a glucose, cPAC+glucose or cPAC-only disk on the MHB-II agar plate. The diameter of the clear zone (CZ) and no colony formation inside the clear zone surrounding the cPAC+glucose disk indicate no re-activation of antibiotic-exposed cells after disk replacement at Step-2 and colonies inside the inhibition zone (indicated by white arrows) after glucose disk replacement indicate re-activation of antibiotic-exposed cells. Disk diameter: 6 mm.

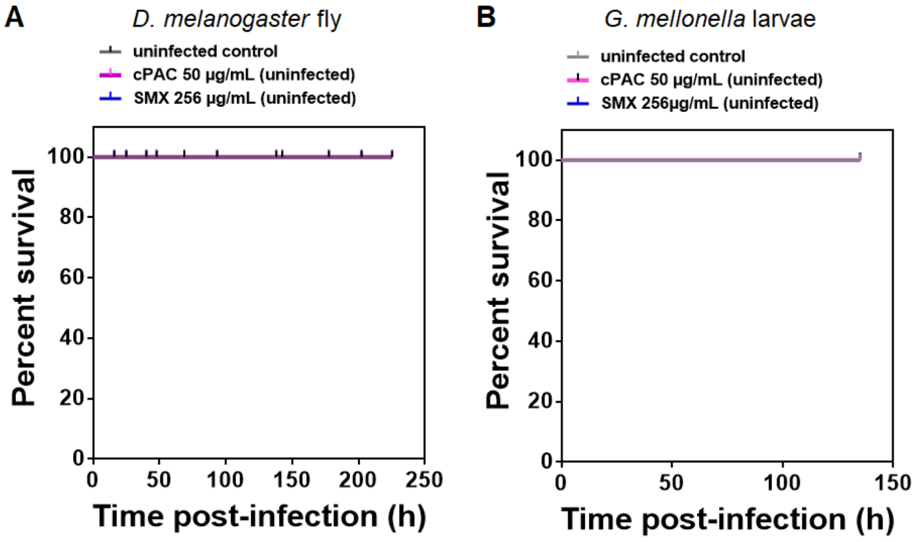

Figure S5. *In vivo* effect of sulfamethoxazole (SMX) or cPAC treatment was tested in (**A**) *D. melanogaster* fly feeding model and (**B**) *G. mellonella* larvae infection model. *D. melanogaster* flies (*n*=30 per experimental group) were infected orally with *P. aeruginosa* PA14 cells and maintained on agar containing SMX 256 µg/mL or cPAC 50 µg/mL alone. Mortality of flies was scored daily for 14 days. Results of the fly model represent measurements from experiments performed with triplicates. *G. mellonella* larvae (*n*=20 per experimental group) were injected with SMX 256 µg/mL or cPAC 50 µg/mL, alone and incubated at 28 °C. Mortality of flies was scored daily for 6 days. Results represent measurements from two independent experiments performed with duplicates.

Table S1. *In silico* molecular docking analysis of efflux pump components and cPAC

	TolC	AcrA	AcrB	OprM[b] (site-1)	OprM[b] (site-2)	MexA	MexB
PDB ID	5v5s	5v5s	5v5s	3d5k	3d5k	2v4d	3w9i
cPAC Topography							
Volume (Å^3)	504.5	490	491.5	508.9	489.5	488.3	506.7
Area (Å^2)	430.5	459.5	461	420.9	461	462.8	423.9
Protein Topography							
Volume (Å^3)	562.7	1538.3	3096	584.8	715.7	1116.9	4403.4
Area (Å^2)	686.8	1179.2	2040.7	573.6	535.7	475.2	2134.6
Autodock Vina binding energy(kcal/mol)	-8	-8.3	-8.2	-9.1	-7.6	-6.8	-9.6
SwissDock server binding energy (kcal/mol)	-7.9	-6.7	-8.1	-7.9	-7.7	-7.1	-8.8
ΔG_{vdw} (kcal/mol)[a]	-48.1	-35.9	-46.3	-44.3	-45.6	-34.3	-59.2
$\Delta G_{lig\ solvpol}$ (kcal/mol)[a]	-23.9	-25.0	-24.6	-23.9	-24.5	-24.3	-24.1
E_{intra} (kcal/mol)[a]	75.2	71.4	76.4	73.3	71.5	75.4	74.1
E_{inter} (kcal/mol)[a]	-48.1	-35.9	-46.3	-44.3	-45.6	-34.3	-59.2
$\Delta G_{lig\ solvnonpol}$ (kcal/mol)[a]	12.3	12.5	12.3	12.1	12.5	12.1	12.4
Full Fitness Score	-1916.0	-1264.4	-4441.8	-2000.8	-1997.7	-1344.7	-3702.9

[a] ΔG_{vdw}, Vander wall or Lennard–Jones potential; $\Delta G_{lig\ solvpol}$, charge-dependent variant of volume-based ligand atomic solvation; E_{intra}, internal ligand energy between atoms of the ligand (electrostatic, steric and hydrogen interactions); E_{inter}, ligand-protein interaction energy; $\Delta G_{lig\ solvnonpol}$, charge-independent variant of volume-based ligand atomic solvation

[b] Binding site-1 with 0 RMSD u.b. (upper bound) and 0 RMSD l.b. (lower bound); Binding site-2 with 15.8 RMSD u.b. and 19.3 RMSD l.b. obtained from Autodock Vina docking analysis.

RMSD u.b. matches each atom in one conformation with itself in the other conformation, ignoring any symmetry. RMSD' matches each atom in one conformation with the closest atom of the same element type in the other conformation (RMSD' can not be used directly, because it is not symmetric). RMSD l.b. is defined as follows: RMSD l.b.(c1, c2) = max [RMSD'(c1, c2), RMSD'(c2, c1)]

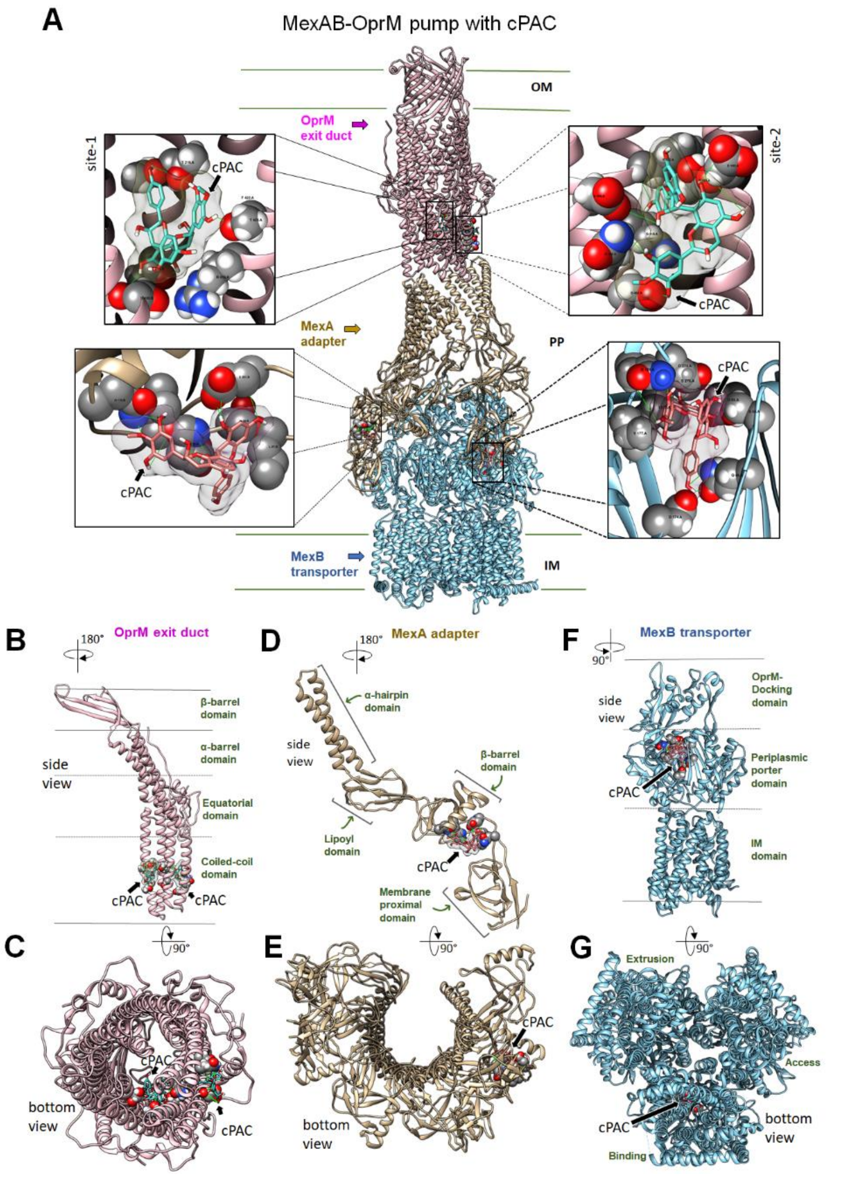

Figure S6. Molecular docking analysis of the MexAB-OprM efflux pump with the inhibitor cPAC.(**A**)The top panel shows full side view with ribbon representation of docked complexes of efflux pump proteins with A-type cPAC molecule (a dimeric form of epicatechin), visualized along the gram-negative cell membrane plane (OM, outer membrane; PP, periplasmic space; IM, inner membrane). The inset views show the inhibitor-binding sites of multidrug efflux pump exit duct, adapter and transporter proteins, indicated with black arrows. The electron density map ($2F_0$-F_c) of cPAC is shown in the hydrophobic trap of each protein structure (inset views). The amino acid residues around this binding site are depicted using the space-filling sphere model and all possible hydrogen bonds are shown using green lines. The ribbon representation of tripartite efflux pump components are color-coded: OprM exit duct (pink), MexA adapter (golden) and MexB transporter (blue). The location of the cPAC binding cavities at different domains during in silico docking are indicated with black arrows and visualized in monomeric side views of (**B**) chain-A of OprM, (**D**) chain-D of MexA and (**F**) chain-J of MexB. The bottom panel represents top views of (**C**) OprM exit duct trimer, (**E**) MexA adapter hexamers, and (**G**) MexB transporter trimer docked complexes with cPAC molecule.

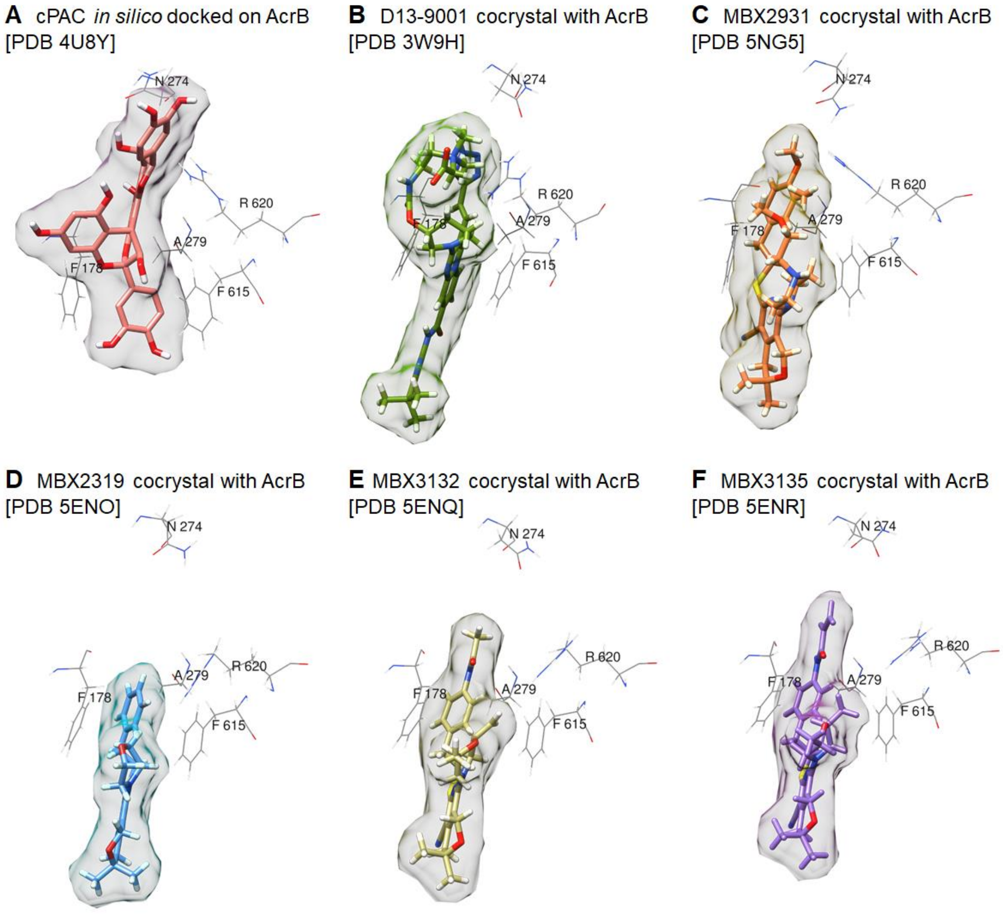

Figure S7. Comparison of cPAC binding position with that of known efflux pump inhibitors in co-crystal AcrB structures.(**A**) The position of cPACwith optimum binding energy at the distal substrate binding pocket in AcrB obtained from *in silico* analysis. (**B**-**F**) Positions of the various inhibitor ligands found in X-ray co-crystal structures with respect to the distal substrate binding pocket in AcrB. The ligands are shown in thick sticks colored according to the atom type (red, oxygen; yellow, sulfur; dark blue, nitrogen; white, hydrogen), the density of the ligands is shown in the hydrophobic trap of AcrB and the representative residues are labeled and shown as wire models.

References

[1] Clinical and Laboratory Standards Institute Methods for Dilution Antimicrobial Susceptibility Tests for Bacteria That Grow Aerobically. CLSI document M07-A8, Clinical and Laboratory Standards Institute: Wayne, PA. Approved Standard, 7th ed. ***2009***.

[2] F. C. Odds, *J. Antimicrob. Chemother.* **2003**, *52*, 1.

[3] E. I. Lutter, M. M. Faria, H. R. Rabin, D. G. Storey, *Infect. Immun.* **2008**, *76*, 1877.

[4] Y. Apidianakis, L. G. Rahme, *Nat. Protoc.* **2009**, *4*, 1285.

[5] G. Jander, L. G. Rahme, F. M. Ausubel, *J. Bacteriol.* **2000**, *182*, 3843.

[6] S. Miyata, M. Casey, D. W. Frank, F. M. Ausubel, E. Drenkard, *Infect. Immun.* **2003** 71, 2404.

[7] J. Rosenblatt, R. Reitzel, T. Dvorak, Y. Jiang, R. Y. Hachem, I. I. Raad, *Antimicrob. Agents Chemother.* **2013**, *57*, 3555.

[8] M. Okshevsky, R. L. Meyer, *J. Microbiol. Methods* **2014**, *105*, 102.

[9] L. Boulos, M. Prevost, B. Barbeau, J. Coallier, R. Desjardins, *J. Microbiol. Methods.* **1999**, *37*, 77.

[10] J. J. Hilliard, R. M. Goldschmidt, L. Licata, E. Z. Baum, K. Bush, *Antimicrob. Agents Chemother.* **1999**, *43*, 1693.

[11] A. J. O'Neill, K. Miller, B. Oliva, I. Chopra, *J. Antimicrob. Chemother.* **2004**, *54*, 1127-1129.

[12] O. Trott, A. J. Olson, *J. Comput. Chem.* **2010**, *31*, 455

[13] A. Grosdidier, V. Zoete, O. Michielin, *Nucleic Acids Res.* **2011**,*39*, W270.
